# Structure, Antioxidant and Anti-inflammatory Activities of the (4*R*)- and (4*S*)-epimers of *S*-Carboxymethyl-L-cysteine Sulfoxide

**DOI:** 10.3390/ph13100270

**Published:** 2020-09-25

**Authors:** James K. Waters, Steven P. Kelley, Valeri V. Mossine, Thomas P. Mawhinney

**Affiliations:** 1 Experiment Station Chemical Laboratories, University of Missouri, Columbia, MO 65211, USA; WatersJ@missouri.edu; 2 Department of Chemistry, University of Missouri, Columbia, MO 65211, USA; KelleySP@missouri.edu; 3 Department of Biochemistry, University of Missouri, Columbia, MO 65211, USA; MawhinneyT@missouri.edu

**Keywords:** COPD, carbocisteine, crystal structure, tumor necrosis factor, hydrogen peroxide, hydroquinone, cigarette smoke extract

## Abstract

*S*-Carboxymethyl-L-cysteine (CMC) is an antioxidant and mucolytic commonly prescribed to patients with chronic obstructive pulmonary disease. In humans, CMC is rapidly metabolized to *S*-carboxymethyl-L-cysteine sulfoxide (CMCO). In this study, we assessed structural and functional similarities between CMC and CMCO. X-Ray diffraction analysis provided detailed structural information about CMCO, which exists as a 1:1 mixture of epimers, due to the emergence of a new chiral center at the sulfur atom. Both CMC and CMCO epimers protected model DNA from copper-mediated hydroxyl free radical damage. Using an insulated transposable construct for reporting activity of the cellular stress-responsive transcription factors Nrf2, p53, NF-κB, and AP-1, we demonstrate that CMCO, especially its (4*R*)-epimer, is comparable to CMC in their ability to mitigate the effects of oxidative stress and pro-inflammatory stimuli in human alveolar (A549) and bronchial epithelial (BEAS-2B) cells. The results of these in vitro studies suggest that CMCO retains, at least partially, the antioxidant potential of CMC and may inform pharmacodynamics considerations of CMC use in clinics.

## 1. Introduction

Chronic obstructive pulmonary disorder (COPD) is a serious life-threatening disease that affects over 325 million people globally, with estimated 6% of all deaths due to this malady [1]; only cardiovascular disease, cancer, and stroke kill more. COPD is primarily caused by tobacco smoke and is characterized by persistent pro-inflammatory responses in the respiratory tract, upon which the airway cells are subjected to a chronic oxidative stress [2]. Though COPD is not curable, a number of therapies, including treatment of exacerbations with mucolytic antioxidant agents, were offered to attenuate damaging effects of the chronic inflammation in lungs of COPD patients. *S*-Carboxymethyl-L-cysteine (CMC, carbocisteine) is an antioxidant and a mucolytic commonly prescribed to patients with COPD [3]. Although CMC has a long history of a clinical drug use, CMC metabolism has been a subject of debate for a number of years [4]. Sulfoxidation is believed to be one of the main metabolic transformations of CMC, with hepatic phenylalanine 4-mono-oxygenase playing a major role in CMC oxidation [5]. To the best of our knowledge, the functional properties of the sulfoxidation product, *S*-carboxymethyl-L-cysteine sulfoxide (CMCO), related to mucolytic/antioxidant/anti-inflammatory activities of CMC have not been evaluated.

**Scheme 1:**
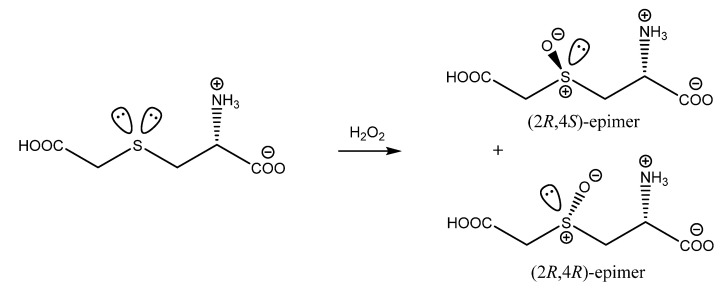
Formation of *S*-carboxymethyl-L-cysteine sulfoxide (CMCO) through mild oxidation of *S*-Carboxymethyl-L-cysteine (CMC).

Structure of CMCO has been deduced from an X-ray diffraction study in 1976 [6]. The reported structure represented only one out of two possible isomers that should form as a result of an emerging new chiral center at the sulfur atom in CMCO, upon mild oxidation of CMC (Scheme 1). In addition, one unusual feature of the reported structure was an assignment of the carboxylate proton to the α-carboxylic group, at an expense of deprotonated δ-carboxylic group. Given that acidity of the α-carboxylic groups in acidic α-amino acids, including CMC and CMC sulfone, is expected to be the highest [7,8,9,10,11], the accuracy of the carboxylic proton assignment in CMCO needs to be re-assessed.

Thus, the main objectives of this work were to re-investigate the structure of *S*-carboxymethyl-L-cysteine sulfoxide and to compare the antioxidant and anti-inflammatory potential of CMC and CMCO in an in vitro model of lower airways using established pathway reporter cell lines.

## 2. Results

*S*-Carboxymethyl-L-cysteine sulfoxide was readily formed by oxidation of CMC with cold 30% hydrogen peroxide (Scheme 1). The ion-exchange chromatographic analysis revealed the presence of two products in a 1:1 ratio (Supplementary Figure S1). Fractional crystallization afforded three crops of CMCO solid with different crystal parameters, all containing pure epimers of CMCO. The epimeric purity of the crops has been established by chromatography and polarimetry, and the absolute structures of the CMCO epimers were further confirmed by the X-ray diffraction analysis data, which are presented below.

### 2.1. Molecular and Crystal Structures of CMCO Epimers

#### 2.1.1. Polymorphs of the (2*R*,4*R*)-*S*-carboxymethylcysteine sulfoxide epimer

**Figure 1:**
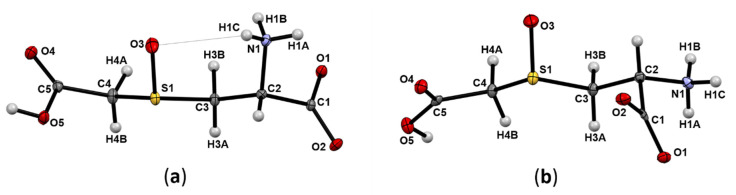
Atom numbering and displacement ellipsoids at 50% probability level for (2*R*,4*R*)-*S*-carboxymethylcysteine sulfoxide polymorphs crystallized in: (**a**) the triclinic space group; (**b**) the orthorhombic space group. An intramolecular hydrogen bond is shown as a thin dotted line.

Sequential crystallization of the CMCO epimeric mixture from warm (45 °C) and cold (0 °C) aqueous solutions afforded crystal crops of the (2*R*,4*R*)-*S*-carboxymethylcysteine sulfoxide ((4*R*)-CMCO), which we determined as belonging to the triclinic *P*1 (structure referred to as t-(4*R*)-CMCO) and orthorhombic *P*2_1_2_1_2_1_ (structure referred to as o-(4*R*)-CMCO) space groups, respectively (Supplementary Table S1), thus demonstrating existence of crystalline polymorphs for this molecule. Further low-temperature X-ray diffraction analysis data (Supplementary Tables S2–S9) has confirmed that, in these two types of crystals, (4*R*)-CMCO molecules assume different conformations (Figure 1).

In both structures, the amino acid molecules exist as zwitterions, with the deprotonated α-carboxylic group and the protonated α-amino- and δ-carboxylic groups; a similar arrangement was found for both CMC [10] and CMC sulfone [9] molecules in the crystalline state. The main difference in the conformations of t-(4*R*)-CMCO and o-(4*R*)-CMCO is due to a rotation around the C2–C3 bond. Thus, in t-(4*R*)-CMCO, atoms S1 and C1 are in the antiperiplanar conformation, with the torsion angle S1-C3-C2-C1 at 170.34°, while in o-(4*R*)-CMCO, the conformation around the C2–C3 bond is *gauche* in respect to the atoms S1 and C1, with the corresponding torsion angle at 66.36°. The conformation displayed by CMC sulfone in crystallo [9] is nearly similar to t-(4*R*)-CMCO, while structure of the CMC molecules [10] differs from o-(4*R*)-CMCO by a single rotation around the S1–C3 bond. In addition, t-(4*R*)-CMCO features a suspected intramolecular hydrogen bond between the amino group and the sulfoxide oxygen atom O3 (Supplementary Table S5). A weak intramolecular H-bond including one ammonium hydrogen and S1 is assumed in the crystalline CMC as well. In o-(4*R*)-CMCO, there is an indication of a strong hydrogen bond between the δ-carboxylate O5 and the α-carboxylate O2, with an unusually short distance between proton H5 and its acceptor O2, such that the O5–H5 bond in o-(4*R*)-CMCO is considerably longer than a similar bond in CMC and other CMCO molecules (1.15 Å in o-(4*R*)-CMCO versus 0.89 Å in t-(4*R*)-CMCO, 0.82 Å in m-(4*S*)-CMCO, and 0.98 Å in CMC, Table 1). In addition, delocalization of the π-bonding in the α-carboxylic group at C5 is higher in o-(4*R*)-CMCO, as compared to the similar reference structures, so that the length difference between C5–O4 and C5–O5 bonds in o-(4*R*)-CMCO is significantly shorter (0.055 Å in o-(4*R*)-CMCO versus 0.08–0.1 Å in the rest). These parameters are in agreement with the previously published orthorhombic (4*R*)-CMCO [6] structure, as well (Table 1).

#### 2.1.2. The (2*R*,4*S*)-*S*-carboxymethylcysteine sulfoxide epimer

**Figure 2:**
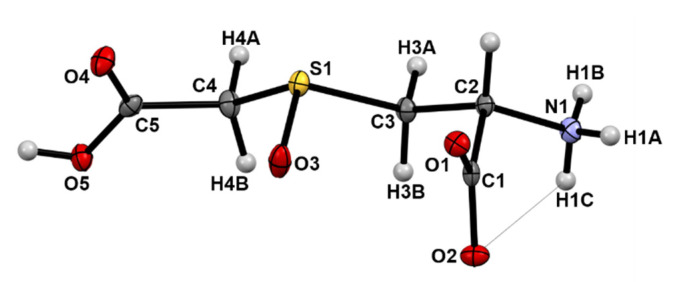
Atom numbering and displacement ellipsoids at 50% probability level for (2*R*,4*S*)-*S*-carboxymethylcysteine sulfoxide crystallized in the monoclinic space group. A suspected intramolecular hydrogen bond is shown as a thin dotted line.

Although (2*R*,4*S*)-*S*-carboxymethylcysteine sulfoxide ((4*S*)-CMCO) was initially identified in a crop of a co-crystallized mixture of the (4*S*)- and (4*R*)-CMCO epimers, crystals containing this molecule and suitable for X-ray diffraction studies were obtained in a course of fractional crystallization. These have been identified as crystals of pure (4*S*)-CMCO belonging to the monoclinic *P*2_1_ space group, structure referred to as m-(4*S*)-CMCO. Similarly to t-(4*R*)-CMCO and o-(4*R*)-CMCO, the molecule of m-(4*S*)-CMCO (Figure 2, Supplementary Tables S10–S12) exists as a zwitterion, with charged α-carboxylic and α-amino groups. The conformation of m-(4*S*)-CMCO is close to the conformation of o-(4*R*)-CMCO, less position of the sulfoxide oxygen O3. In addition, m-(4*S*)-CMCO features an intramolecular heteroatom interaction between O2 and N1, which may qualify for a weakly directional hydrogen bond, with H1C ··· O2 distance 2.57(3) Å and N1–H1C···O2 angle 95.1(4)°.

#### 2.1.3. Crystal Packing and Intermolecular Hydrogen Bonding

The crystal packing in t-(4*R*)-CMCO, o-(4*R*)-CMCO, and m-(4*S*)-CMCO is shown in Supplementary Figures S2–S4. Since CMC, its sulfoxides, and sulfone are heteroatom-rich, zwitterionic molecules, the contacts involving electronegative heteroatom O dominate in the crystal structures of these molecules, as summarized in Table 2. The H ··· O contacts are responsible for an extensive three-dimensional hydrogen bonding network in all three crystal structures (Supplementary Tables S5, S9, S13; Supplementary Figures S2–S4).

**Figure 3:**
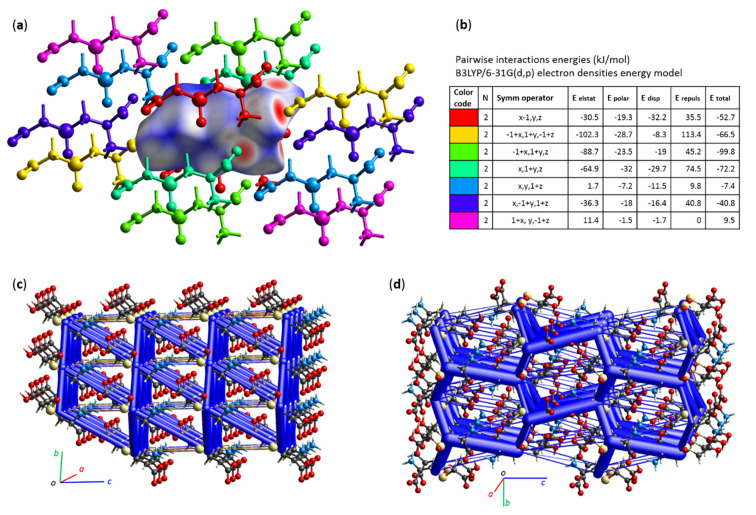
Interaction energies in crystal structures. (**a**) A view of interactions between a central molecule of (4*R*)-CMCO in t-(4*R*)-CMCO, shown as its Hirshfeld surface, and 14 molecules that share the interaction surfaces with the central molecule. (**b**) Calculated energies (electrostatic, polarization, dispersion, repulsion, and total) of pairwise interactions in t-(4*R*)-CMCO between the central molecule and those indicated by respective colors. (**c**) Energy framework for total pairwise interaction energies in t-(4*R*)-CMCO. The cylinders link molecular centroids, and the cylinder thickness is proportional to the magnitude of the energies, such as those shown in (**b**). For clarity, the cylinders corresponding to energies <5 kJ/mol are not shown. (**d**) The total pairwise interaction energy framework in o-(4*R*)-CMCO. For interaction energies in o-(4*R*)-CMCO and m-(4*S*)-CMCO, also see Supplementary Figures S5–S6. For energy frameworks depicting electrostatic and dispersion energies in crystals of CMCO, see Supplementary Figures S7–S9.

Molecular modeling calculations, which were performed for these crystal structures, have confirmed that the electrostatic interactions are pervasive and largely define energetics of the crystal packing in CMC, *S*-carboxymethyl-L-cysteine sulfone (CMCO2), as well as all three CMCO structures (Figure 3, Table 3, Supplementary Figures S5–S9).

### 2.2. CMC and CMCO Protect DNA from Copper-Catalyzed Degradation by Hydroxyl Free Radicals

**Figure 4:**
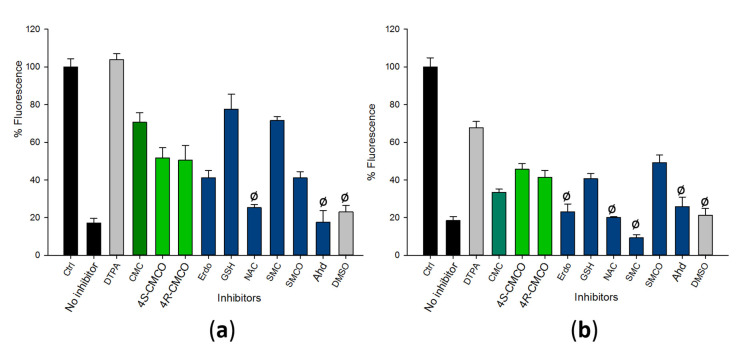
CMC and CMCO protect dsDNA degradation in presence of (**a**) 50 μM copper/2 mM ascorbate/2 mM H_2_O_2_; (**b**) 50 μM copper/4 mM hydroquinone ROS generating systems. Abbreviations: Ctrl, no ROS; DTPA, diethylenetriaminepentaacetic acid; Erdo, erdosteine; GSH, reduced glutathione; NAC, *N*-acetyl-L-cysteine; SMC, *S*-methyl-L-cysteine; SMCO, *S*-methyl-L-cysteine sulfoxide; Ahd, L-2-aminohexanedioic acid. Concentrations of inhibitors—1 mM; DMSO—0.5% *v/v*. For each treatment, *n* = 3. The differences of means were probed by the one-way ANOVA; ø indicates no statistically significant difference between a treatment and “no inhibitor” control; otherwise, *p* < 0.001.

Redox-active metals, such as copper, and aromatic molecules, such as hydroquinone, from cigarette smoke can promote cellular nucleic acids damage by reactive oxygen species (ROS) and are thought to contribute to development of COPD and other pulmonary disorders in smokers [2,13,14,15]. We have compared the protective effects of CMC and CMCO as antioxidants in a conventional in vitro model of DNA degradation by hydroxyl free radicals generated in the copper-catalyzed Fenton reaction. Both epimeric forms of CMCO, along with CMC and their decarboxylated structural analogues, *S*-methyl-L-cysteine and *S*-methyl-L-cysteine sulfoxide, protected DNA from oxidative degradation by hydroxyl free radicals in the copper(II)/hydrogen peroxide/ascorbate system (Figure 4). In contrast, *N*-acetyl-L-cysteine and L-2-aminohexanedioic acid (L-α-aminoadipic acid), a structural analogue of CMC lacking the thioether atom, were not effective. Moreover, CMCO inhibited the oxidative degradation of DNA in the copper(II)/hydroquinone system somewhat more efficiently than CMC. Likewise, *S*-methyl-L-cysteine sulfoxide appeared to act as a better DNA protector than *S*-methyl-L-cysteine in this system (Figure 4).

### 2.3. Effects of CMC and CMCO on Activation of Oxidative Stress and Pro-inflammatory Signalling Pathways in Human Bronchial Epithelial and Alveolar Cell Lines

**Figure 5:**
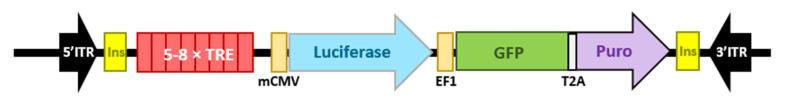
The construct for a transcriptional activity reporter. Abbreviations: TRE, transcriptional response elements; mCMV, minimal CMV promoter; EF1, elongation factor 1α promoter; GFP, green fluorescent protein; Puro, puromycin resistance gene; Ins, insulators; ITR, inverted terminal repeats.

To ascertain potential inhibitory activities of CMCO against COPD-relevant inducers of inflammation and oxidative stress in respiratory cells, we have generated a series of reporters of transcriptional activity of the following four stress-inducible transcriptional factors: p53, Nrf2, NF-κB, and AP-1 [16,17]. A reporter, schematized in Figure 5, represents a DNA sequence containing 5-8 concatenated binding sites for a specific transcription factor, followed by the minimal CMV promoter, a luciferase gene, the EF1 promoter, and the copepod GFP and a selector antibiotic resistance gene. This assembly is flanked by a pair of insulator sequences, which deter epigenetic silencing of the reporter sequence within the cellular host DNA; furthermore, a pair of the *piggyBac* transposon sequences outside of the insulators provides for an efficient integration of the reporter sequence into the host chromatin within regions of enhanced transcriptional activity. Such constructs provided for exceptional stabilities and high functional activities of the transgenes [16]. For this work, we have stably transfected human bronchial epithelial BEAS-2B and human alveolar A549 cell lines with the reporter plasmids; the resulting reporter cell lines thus allowed for conducting the testing experiments in a medium-throughput format.

#### 2.3.1. The p53 Pathway

**Figure 6:**
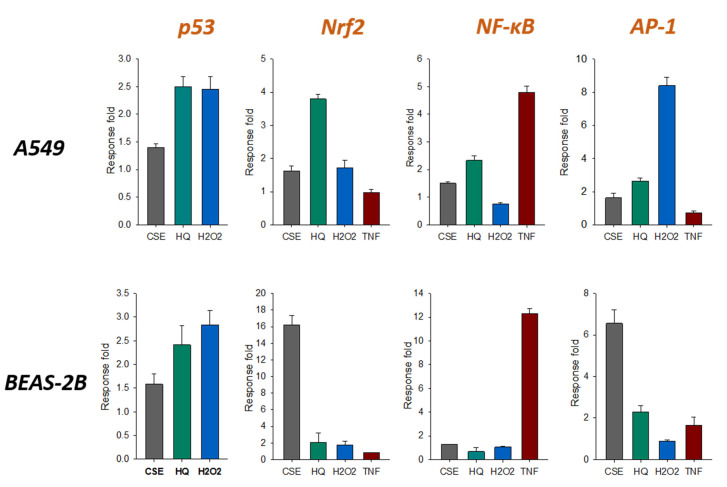
Induction of transcriptional activities of selected TFs in A549 and BEAS-2B cells by a cigarette smoke extract (CSE), hydroquinone (HQ), hydrogen peroxide, and Tumor Necrosis Factor (TNF). The response fold is measured as a ratio of luciferase activities in the treated and untreated cells after 18 h of exposure. The stressor concentrations are 2.5% CSE in both lines; 125 and 32 μM HQ in A549 and BEAS-2B, respectively; 1000 and 125 μM H_2_O_2_ in A549 and BEAS-2B, respectively; 4 ng/mL TNF in both lines. The error bars are SDs; *n* = 3.

One of the main functions of the p53 transcription factor is to respond to cellular DNA damage through regulation of the DNA repair and the cell fate [18]. When A549 and BEAS-2B cell reporters for the p53 pathway were exposed to a diluted (2.5%) cigarette smoke extract for 18 h, the transcriptional activity of the p53 increased by about 50% in both cell lines (Figure 6). Previous studies demonstrated that hydroquinone, a major component of cigarette smoke, could be genotoxic in pulmonary cells [14] via induction of ROS, such as hydrogen peroxide. In our research, both hydroquinone and H_2_O_2_ promoted an increase in the p53 transcriptional activity in both A549 and BEAS-2B cells (Figure 6).

**Figure 7:**
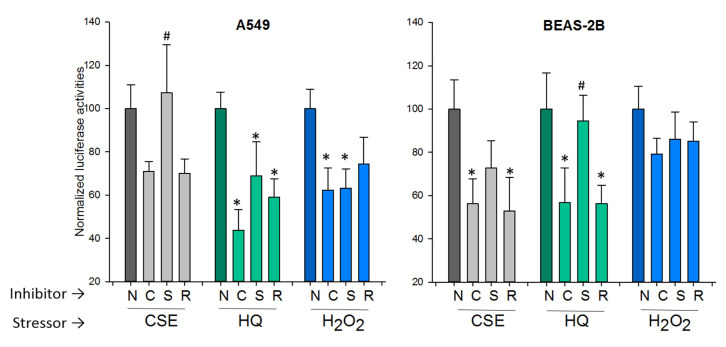
Protection of A549 and BEAS-2B cells from stressors-activators of the p53 pathway by CMC (abbreviated as C), (4*S*)-CMCO (S), and (4*R*)-CMCO (R). The luciferase activities in the treated cells are normalized for the reporter activity in wells receiving no inhibitor (N). The stressor concentrations are indicated in Figure 6. The inhibitor concentrations were 1 mM in all experiments. The error bars are SDs; *n* = 3. The differences of means were probed by the one-way ANOVA; * indicates a statistically significant difference between an inhibitor treatment and “no inhibitor” control; # indicates a statistically significant difference between CMC and CMCO treatments, *p* < 0.05.

In cultured alveolar and bronchial epithelial cells, both CMC and CMCO protected cellular DNA from damage by hydroquinone or cigarette smoke extract, as evidenced by a decrease in activity of the p53 pathway signaling in the antioxidant-treated cells. As demonstrated in Figure 7, CMC and the (4*R*)-CMCO epimer consistently inhibited p53 activation in CSE- and HQ-treated cells, although the effect was less significant in the H_2_O_2_-treated cells. The (4*S*)-CMCO epimer, however, was ineffective or less effective than CMC, in these experiments.

#### 2.3.2. The Nrf2 Pathway

Widely acknowledged as a master regulator of cellular stress, the Nrf2 transcription factor normally responds to an increase in intracellular concentrations of thiol-reactive species, such as ROS, heavy metals, hydroquinones, and unsaturated or carbonyl intermediates [19]. Cigarette smoke contains a large number of these potential inducers for Nrf2 [20]. In most cells, the SH-sensor is represented by the Kelch-like ECH-associated protein 1 (KEAP1), which, in its native state, binds to Nrf2 and directs it to degradation. Electrophiles dysfunction KEAP1 and thus promote survival of Nrf2 and its translocation from the cytoplasm to the nucleus, where it becomes transcriptionally active. A549 cells represent a line with mutated KEAP1 and thus with enhanced basal Nrf2 activity and resistance to oxidative stress [21]. Indeed, as shown in Figure 6, the Nrf2 reporter is considerably more responsive to CSE in BEAS-2B cells, as compared to A549.

**Figure 8:**
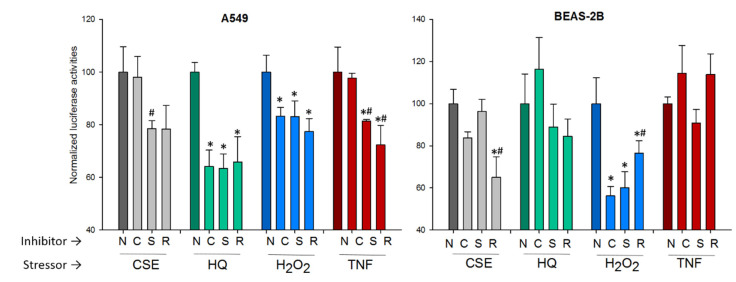
Effects of CMC (C), (4*S*)-CMCO (S), and (4*R*)-CMCO (R) on activation of the Nrf2 pathway by stressors in A549 and BEAS-2B cells. The luciferase activities in the treated cells are normalized for the reporter activity in wells receiving no inhibitor (N). The stressor concentrations are indicated in Figure 6. The inhibitor concentrations were 1 mM in all experiments. The error bars are SDs; *n* = 3. The differences of means were probed by the one-way ANOVA; * indicates a statistically significant difference between an inhibitor treatment and “no inhibitor” control; # indicates a statistically significant difference between CMC and CMCO treatments, *p* < 0.05.

In the presence of CMC and CMCO, there was a significant decrease in the Nrf2 activity in A549 cells treated with H_2_O_2_ or hydroquinone (Figure 8). In addition, CMCO, rather than CMC, inhibited Nrf2 activation in A549 cells treated with CSE or TNF. In BEAS-2B cells, the protective effect of CMC and CMCO was the most pronounced against the H_2_O_2_ stress, while there was no significant influence on the Nrf2 activation by HQ and TNF by any of the inhibitors.

#### 2.3.3. The NF-κB Pathway

In both A549 and BEAS-2B cells, this pro-inflammatory pathway was, as expected, significantly activated by pro-inflammatory cytokine TNF (Figure 6). In both cell lines, treatment with CSE resulted in a moderate (about 50%) increase of the NF-κB activation, as well, while HQ in BEAS-2B and H_2_O_2_ in both lines were not inducing this pathway.

**Figure 9:**
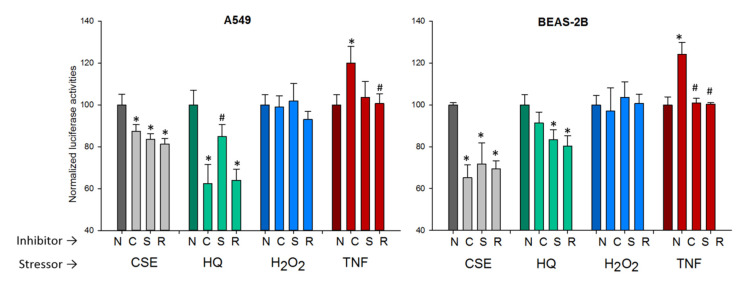
Effects of CMC (C), (4*S*)-CMCO (S), and (4*R*)-CMCO (R) on activation of NF-κB pathway by stressors in A549 and BEAS-2B cells. The luciferase activities in the treated cells are normalized for the reporter activity in wells receiving no inhibitor (N). The stressor concentrations are indicated in Figure 6. The inhibitor concentrations were 1 mM in all experiments. The error bars are SDs; *n* = 3. The differences of means were probed by the one-way ANOVA; * indicates a statistically significant difference between an inhibitor treatment and “no inhibitor” control; # indicates a statistically significant difference between CMC and CMCO treatments, *p* < 0.05.

Both CMC and CMCO could reduce NF-κB activation in cells treated with either CSE or HQ (Figure 9) but had no effect on cells receiving H_2_O_2_. Interestingly, there was a small but significant increase in the NF-κB transcriptional activity in both A459 and BEAS-2B cells treated simultaneously with TNF and CMC, but no interaction between TNF and CMCO was detected in these cells.

#### 2.3.4. The AP-1 Pathway

The Activator Protein-1 pathway is a pro-inflammatory signaling pathway that is activated under conditions of increased intracellular oxidative stress [22]. Indeed, all four stressors could induce significant activation of this pathway in both A549 and BEAS-2B cells, as evidenced by data shown in Figure 6. However, the reporter responses to particular stressors differed between these cell lines. Thus, BEAS-2B was significantly more sensitive to CSE, as compared to A549, while the reporter lines responses to H_2_O_2_ were in the reverse order.

**Figure 10:**
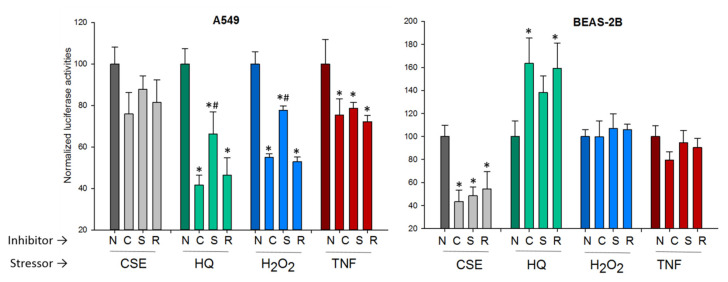
Effects of CMC (C), (4*S*)-CMCO (S), and (4*R*)-CMCO (R) on activation of AP-1 by stressors in A549 and BEAS-2B cells. The luciferase activities in the treated cells are normalized for the reporter activity in wells receiving no inhibitor (N). The stressor concentrations are indicated in Figure 6. The inhibitor concentrations were 1 mM in all experiments. The error bars are SDs; *n* = 3. The differences of means were probed by the one-way ANOVA; * indicates a statistically significant difference between an inhibitor treatment and “no inhibitor” control; # indicates a statistically significant difference between CMC and CMCO treatments, *p* < 0.05.

Both CMC and CMCO could reduce activation of the AP-1 pathway in A549 cells challenged with the stressors, but in BEAS-2B cells, such effect was observed only against CSE (Figure 10). Surprisingly, both CMC and CMCO did enhance the pro-inflammatory effect of HQ in BEAS-2B reporters of the AP-1 pathway.

## 3. Discussion

This work was aimed at establishing whether there are structural and functional similarities between antioxidant drug CMC and its main product of metabolic oxidation in humans, CMCO, because CMCO is considered functionally inactive due to a partial oxidation of the thioether sulfur [23]. This suggestion is based on an indirect observation of CMC efficacy that differed in patients with higher and lower rates of CMC metabolism [4]. However, to the best of our knowledge, no previous clinical, in vivo or in vitro laboratory studies compared efficacy of CMC and CMCO directly.

Non-enzymatic mild oxidation of CMC in vitro brought about a mixture of the (4*R*)- and (4*S*)-*S*-carboxymethyl-L-cysteine sulfoxide that were also detected in humans taking the CMC drug [24]. In vivo, oxidation of circulating CMC is believed to occur primarily in the liver, and no evidence is available to suggest that the oxidation proceeds stereospecifically [4].

Fractional crystallization allowed for complete separation of the CMCO enantiomeric forms. Both (4*R*)- and (4*S*)-CMCO molecules showed structural similarity with CMC, both by conformation and charge distribution. Accordingly, the electrostatic forces defined the energetics of crystal packing in both CMC and CMCO crystal structures. An unusually strong hydrogen bond between α- and δ-carboxylate groups was detected in the orthorhombic crystals of (4*R*)-CMCO, thus confirming an earlier report of this phenomenon [6]. Since we collected the X-ray diffraction data at much lower temperature (100 K) than the previous study (295 K), it allowed us to determine the structure of (4*R*)-CMCO with higher confidence. Our data assigned the proton, which participates in the aforementioned H-bonding, to the δ-carboxylate oxygen O5, as it would be expected for an aliphatic α-aminodicarboxylic acid, based on considerations of the acid-base properties of this type of molecules [7,11].

Next, our data have demonstrated that CMCO, along with CMC, can protect polymeric nucleic acid from degradation by hydroxyl free radicals. Because production of biologically relevant ROS usually involves a process of biomolecules oxidation in presence of a redox-cycling catalyst, such as transition metals copper or iron, successful antioxidants are represented by molecules that inhibit the redox-cycling process. Alternatively, if production of ROS is sufficiently slow, strong reducing agents can quench accumulating ROS, thus preventing oxidative damage to the target of the protection. Both CMC and CMCO can chelate Cu^2+^ ions [25,26] and act as reducing agents due to a presence of the thioether group [27], although potential reducing capacity of the sulfur in CMCO is one-half of that in CMC. Notably, a good copper(II) chelator but poor reducing agent, 2-aminohexanoic acid, as well as an excellent reducing agent but poor copper(II) chelator, *N*-acetyl-L-cysteine, failed to protect DNA from copper-mediated oxidative degradation, in our experiment. In contrast, a versatile intracellular antioxidant, glutathione, which is well known for both its reducing power and copper binding capabilities [28], potently inhibited oxidative degradation of DNA in our test. It should be pointed out that since this experiment was performed outside of a biological milieu, its significance can only be considered as a proof-of-principle for a hypothesis that CMCO can act likewise to CMC as a chemical antioxidant. Biological relevance of similarities in functional activities of CMC and CMCO was further examined in the cell culture.

**Figure 11:**
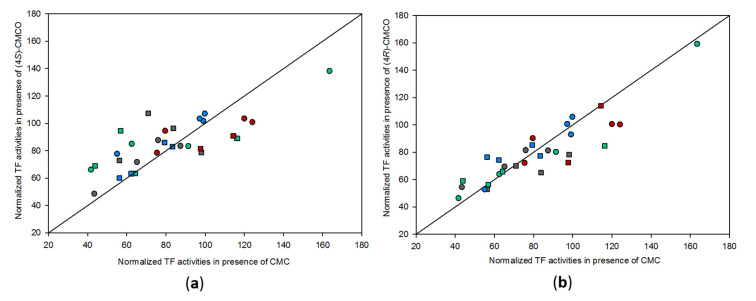
Summarized correlations between effects of: (**a**) CMC and (4*S*)-CMCO; (**b**) CMC and (4*R*)-CMCO on activation of the four transcription factors in A549 (squares) and BEAS-2B (circles) cells, as shown in Figure 7, Figure 8, Figure 9 and Figure 10. Data points depicted below the diagonals signify stronger inhibitory effects displayed by CMCO as compared to CMC. Color codes for the stressors: grey—CSE, green—HQ; blue—H_2_O_2_, red—TNF.

Although CMC is considered a mucolytic, this molecule lacks free thiol groups that are needed to break the S-S bonds in mucus proteins that stabilize the matrix. Rather, CMC is believed to relieve inflammation and decrease mucus secretion in the lower airways [3]. Our study compared the ability of CMC and CMCO to affect cell signaling pathways, which are sensitive to oxidative stress and pro-inflammatory stimuli, in human bronchial epithelial and alveolar cells. As shown in Figure 11, there is generally a good correlation between the activities (or non-activities) displayed by CMC and CMCO in these experiments. However, one can notice (Figure 6, Figure 8 and Figure 9) that there is a substantial difference between the activities of the (4*R*)- and (4*S*)-CMCO epimers, with the former providing significantly more protection against the cell stressors. Such a pattern may hint at stereospecific mechanisms of CMCO utilization by the cells, which could include its reduction to CMC, for example.

Another pattern noticeable in Figure 11 is that CMC performs better than CMCO when protecting cells from CSE- or H_2_O_2_-induced stresses. In contrast, cellular responses to pro-inflammatory TNF in presence of CMC were, in a number of cases, stronger, as compared to the CMCO treatments. In fact, CMC did enhance the NF-κB activation in the TNF-treated cells.

In conclusion, results of this study have demonstrated an existence of some structural and functional similarities between *S*-carboxymethyl-L-cysteine drug and two epimers of its metabolite, *S*-carboxymethyl-L-cysteine sulfoxide. Precise structures of (4*S*)-CMCO and two polymorphs of (4*R*)-CMCO, as well as their proton distribution, conformational and energetic similarities to CMC, were determined. In a DNA oxidative degradation chemical model, CMCO exhibited antioxidant activities that were comparable to CMC. In cell-based models of pulmonary inflammation and oxidative stress, the CMCO protective effects correlated closely with those displayed by CMC, too. Because these results were obtained in vitro only, their significance for clinical use of CMC could not be established. However, these data might be informative for future pharmacodynamics studies of CMC and could contribute methodologically to a wider use of cell signaling reporters to characterize pharmaceuticals, nutraceuticals, or toxic agents.

## 4. Materials and Methods

All commercial reagents and cell culture media were purchased from Fisher (Fair Lawn, New Jersey, NJ, USA) or Sigma-Aldrich (St Louis, Missouri, MO, USA) companies. Cigarette smoke extract (CSE) has been prepared following a simplified protocol [29] with some modifications: the mainstream smoke from four unfiltered cigarettes was bubbled through 4 mL phosphate-buffered saline, pH 7, using a 20 mL syringe pump. CSE was immediately sealed and kept on ice until use.

A549 (passage 81) human alveolar and BEAS-2B (passage 38) human bronchial epithelial cell lines were purchased from the American Type Culture Collection. The cells were maintained in DMEM media supplemented with 5% Newborn Calf Serum and 1% of the penicillin/streptomycin antibiotic cocktail, at 37 °C, 100% humidity, and 5% CO_2_.

Optical rotation data were collected using a Jasco P-1030 polarimeter (JASCO, Tokyo, Japan). Chromatographic analyses were performed with Hitachi (L-8900) amino acid analyzer, using the manufacturer’s lithium buffer system (Hitachi Group, Tokyo, Japan). All fluorescence and luminescence measurements were done using a Synergy MX plate reader (BioTek Instruments, Winooski, Vermont, VT, USA).

### 4.1. Synthesis and Crystallization of S-carboxymethyl-L-cysteine sulfoxide

The synthetic CMCO preparation procedure generally followed a protocol originally developed by Meese [30]. In one experiment, 35.8 g (0.2 moles) of *S*-carboxymethyl-L-cysteine were dissolved in 134 mL water containing 16.8 g NaHCO_3_ and cooled in an ice bath. To the solution were added, dropwise, 40 mL cold 30% H_2_O_2_, and the reaction mixture was left for 20 h at 4 °C. The completeness of the oxidation reaction was confirmed chromatographically and the excess of H_2_O_2_ was destroyed with catalytic amounts of MnO_2_. The reaction mixture was then filtered, acidified with 40 mL 5N HCl, and left for 2 days at 8 °C. A crystalline mass had formed and was separated by filtration. Chromatography and polarimetry data confirmed the formation of a 1:1 mixture of two CMCO epimers; [α]_D_^23^ = +31.4° (c = 1.0, 0.2 N HCl); literature [30] [α]_D_^21^ = +28.2° (c = 1.0, 1 N HCl); nitrogen analysis: 7.74%, calcd for C_5_H_9_NO_4_S: 7.82%. In another experiment, the crystallization step was carried at 45 °C for 16 h. The collected crystals, shaped as blocks and belonging to the triclinic space group, had a specific optical rotation value consistent with the (4*R*)-epimer of *S*-carboxymethyl-L-cysteine sulfoxide: [α]_D_^23^ = +59.4° (c = 1.0, 0.2 N HCl); literature [30] [α]_D_^21^ = +63.7° (c = 1.0, 1 N HCl). The mother liquor was cooled to 0 °C and deposited an additional crop of the (4*R*)-epimer, [α]_D_^23^ = +53.5° (c 1, 0.2 N HCl), in the shape of needles, the orthorhombic space group. The remaining mother liquor contained predominantly (>90%) the (4*S*)-epimer, according to a chromatographic analysis of the sample. This solution was filtered, diluted with an equal volume of ethanol, and kept at 8 °C for 3 days, to deposit monoclinic plates of chromatographically pure (>95%) (4*S*)-epimer, [α]_D_^22^ = +1.1° (c = 1.0, 0.2 N HCl); literature [30] [α]_D_^21^ = −4.2° (c = 1.0, 1 N HCl).

### 4.2. X-ray Diffraction Studies

Crystal data, data collection, and structure refinement details are summarized in Table S1. Oxygen-bound H atoms were located from the difference map and those bonded to carbon were placed in calculated positions. The coordinates of all H atoms were refined freely while the thermal parameters were constrained to ride on the carrier atoms, *U*_iso_(H) = 1.2–1.5 *U*_eq_(C,O). Structure refinement and visualization was done with computer programs SHELXL [31], OLEX2 [32], Mercury [33].

### 4.3. In Vitro DNA Protection

To a 200 μL solution of 50 μg polymeric DNA (from calf thymus, Sigma) per mL of Chelex-treated PBS, pH 7, were added, in order, CMCO or other antioxidants, CuCl_2_ (final concentration 50 μM), H_2_O_2_ (final concentration 2 mM), and ascorbic acid (final concentration 2 mM). Alternatively, hydroquinone (final concentration, 4 mM) was added instead of H_2_O_2_/ascorbate. The reaction was left to proceed at room temperature for 2 h and then stopped by addition of DTPA to a final concentration of 5 mM. Ethidium bromide was added at 20 μg/mL, and the fluorescence of the solutions was measured at 508 nm excitation/590 nm emission wavelengths.

### 4.4. Signaling Pathway Reporters

#### 4.4.1. Reporter Vectors

Super *piggyBac* transposase expression vector PB210PA-1 was purchased from System Biosciences. The preparation and validation of reporter plasmids carrying insulated *piggyBac* transposon constructs, which contain transcriptional response element (TRE) and reporter genes, firefly or Brazilian click beetle luciferase, and a copepod green fluorescent protein, as shown in Figure 5, were reported earlier [16,17,34]. Before transfections, the integrity of plasmids was verified by DNA electrophoresis and sequence analysis.

#### 4.4.2. Stable Transfections

To generate stable reporter lines, the original A549 and BEAS-2B cells were seeded into wells of a 96-well plate, at 2 × 10^4^ cells per well in antibiotic-free DMEM/F12 medium supplemented with 5% NCS and left to adhere for 6 h. The cells were then treated with a mixture of 100 ng of a reporter plasmid and 33 ng of Super piggyBac transposase plasmid complexed with TransIT X2 transfection reagent (Mirus) at 1:2 (μg DNA/ μL) ratios. After 16 h, the regular media were added and the cells were left to proliferate for next 48–72 h. The transfected cells were then treated with the selecting antibiotic (5 μg/mL puromycin) for another week, and the surviving cells were expanded for cryopreservation and activity validation.

#### 4.4.3. Transcriptional Activity Reporter Assay

In a typical experiment, reporter cells were seeded into wells of a 96-well plate, at 1 × 10^4^ cells per well in DMEM/F12 medium supplemented with 5% NCS and penicillin/streptomycin antibiotic and left to proliferate for 48 h. The medium was changed to the experimental medium, Corning Serum-free Medium, which is essentially a Phenol Red-free DMEM/F12 formulation with undisclosed additions of RPMI-1640 and McCoy’s 5A, and is supplemented with 1 g/L BSA, 2 mg/L insulin, 2 mg/L transferrin, and 2 μg/L selenite. After an 18 h adaptation period, the medium was replaced with fresh experimental medium, now containing stressor and inhibitor agents. The experimental treatments lasted 18 h in standard conditions (37 °C, 100% humidity and 5% CO_2_). The cells were then lysed in 70 μL of the Luciferase reporter lysing buffer (Promega). GFP content in the lysates was determined by fluorescence at the 482(9)/512(17) nm wavelength (slit width) setup; this was followed by an addition of the luciferase substrate (Promega) and luminescence readings in the wells. The GFP fluorescence values were used for both evaluation of relative cell transcriptional activity/proliferation and normalization of the reporter luciferase activities in respective wells [16].

### 4.5. Molecular Modeling and Statistical Analysis

The Hirshfeld surfaces analyses and DFT calculations, at the B3LYP/6-31G(d,p) theory level, were performed using CrystalExplorer software (version 17.5) [12,35]. Statistical tests and plots were done by using SigmaPlot (version 11.0).

## Figures and Tables

**Table 1:** Comparative geometries (Å, °) of carboxylate-carboxylate hydrogen bonds in CMCO and related structures.

Molecule	Space Group	*D*–H ··· *A*	*D–H*	H ··· *A*	*D* ··· *A*	*D*–H ··· *A*	Δ_C1–O_^1^	Δ_C5–O_^2^
t-(4*R*)-CMCO	Triclinic	O5–H ··· O1	0.89 (2) ^3^	1.64 (3)	2.500 (1)	163 (2)	0.018	0.083
o-(4*R*)-CMCO	Orthorhombic	O5–H ··· O1	1.15 (3)	2.44 (3)	3.247 (2)	126 (2)	0.039	0.055
		O5–H ··· O2	1.15 (3)	1.32 (3)	2.450 (2)	168 (3)		
m-(4*S*)-CMCO	Monoclinic	O5–H ··· O2	0.82 (4)	1.67 (4)	2.489 (3)	176 (4)	0.010	0.083
CMC [10]	Monoclinic	O5–H ··· O2	0.98 (3)	1.58 (3)	2.548 (2)	169 (3)	0.008	0.102
(4*R*)-CMCO [6]	Orthorhombic	O2–H ··· O5	1.04 (4)	1.41 (4)	2.449 (3)	176 (4)	0.032	0.058
CMCO2 [9]	Orthorhombic	O5–H ··· O2	0.96 (5)	1.54 (5)	2.504 (5)	175 (5)	0.048	0.098

^1^ Length difference between C1–O1 and C1–O2 bonds, Å. ^2^ Length difference between C5–O4 and C5–O5 bonds, Å. ^3^ The estimated standard deviation of the last digit.

**Table 2:** Contributions (%) of the intermolecular contacts to the Hirshfeld surfaces [12] of *S*-carboxymethyl-L-cysteine and its oxidation products.

Molecule	H ··· O	H ··· H	H ··· S	H ··· C	O ··· O	O ··· S	O ··· C	C ··· C	S ··· C
CMC [10]	58.5	22.2	9.1	2.2	2.5	2.9	0.6	0.6	1.5
t-(4*R*)-CMCO	68.8	18.1	4.6	0.6	2.4	2.6	2.9	0	0
o-(4*R*)-CMCO	77.6	10.3	4.9	2.9	2.0	0.7	1.6	0	0
m-(4*S*)-CMCO	68.4	18.2	4.9	0.4	2.8	2.0	2.4	0	0.9
CMCO2 [9]	78.4	11.2	0	1.4	5.8	0	3.2	0	0

**Table 3:** Contributions of intermolecular interactions to the packing energies **E** (kJ/mol) in crystalline structures of *S*-carboxymethyl-L-cysteine and its oxidation products.

Structure	E Electrostatic	E Polar	E Dispersion	E Repulsion	E Total
CMC [10]	−302.7	−124.6	−103.4	277.3	−331.1
t-(4*R*)-CMCO	−309.6	−130.2	−118.8	319.2	−329.9
o-(4*R*)-CMCO	−336.8	−148.7	−117.3	350.2	−351.8
m-(4*S*)-CMCO	−323.4	−157.7	−118.7	318.4	−365.6
CMCO2 [9]	−335.7	−142.4	−126	319.7	−372.6

## Supplementary Materials

The following are available online at https://www.mdpi.com/1424-8247/13/10/270/s1, Figure S1: Ion-exchange chromatograms of CMCO crystalline forms; Figure S2: The molecular packing in t-(4*R*)-CMCO; Figure S3: The molecular packing in o-(4*R*)-CMCO; Figure S4: The molecular packing in m-(4*S*)-CMCO; Figure S5: Interaction energies in crystal structure of o-(4*R*)-CMCO; Figure S6: Interaction energies in crystal structure of m-(4*S*)-CMCO; Figure S7: Energy framework for pairwise interactions in t-(4*R*)-CMCO; Figure S8: Energy framework for pairwise interactions in o-(4*R*)-CMCO; Figure S9: Energy framework for pairwise interactions in m-(4*S*)-CMCO; Table S1: Crystal data, data collection, and structure refinement details; Table S2: Fractional atomic coordinates and isotropic or equivalent isotropic displacement parameters for t-(4*R*)-CMCO; Table S3: Atomic displacement parameters for t-(4*R*)-CMCO; Table S4: Bond distances and angles for t-(4*R*)-CMCO; Table S5: Hydrogen-bond geometry t-(4*R*)-CMCO; Table S6: Fractional atomic coordinates and isotropic or equivalent isotropic displacement parameters for o-(4*R*)-CMCO; Table S7: Atomic displacement parameters for o-(4*R*)-CMCO; Table S8: Bond distances and angles for o-(4*R*)-CMCO; Table S9: Hydrogen-bond geometry o-(4*R*)-CMCO; Table S10: Fractional atomic coordinates and isotropic or equivalent isotropic displacement parameters for m-(4*S*)-CMCO; Table S11: Atomic displacement parameters for m-(4*S*)-CMCO; Table S12: Bond distances and angles for m-(4*S*)-CMCO; Table S13: Hydrogen-bond geometry m-(4*S*)-CMCO. The complete crystallographic data for the structural analysis have been deposited with the Cambridge Crystallographic Data Centre, CCDC ## 2027234, 2027235, 2027236. Copies of this information may be obtained free of charge from the Director, Cambridge Crystallographic Data Centre, 12 Union Road, Cambridge, CB2 1EZ, UK. (Fax: +44-1223-336033, e-mail: deposit@ccdc.cam.ac.uk or via: www.ccdc.cam.ac.uk).
