# Supplementary material for: Structure, Antioxidant and Anti-inflammatory Activities of the (4R)- and (4S)-epimers of S-Carboxymethyl-L-cysteine Sulfoxide
Source: Pharmaceuticals (Basel). 2020 Sep 25;13(10):270. doi: 10.3390/ph13100270 (PMC7600183; doi:10.3390/ph13100270)
Supplement: Supplementary file 1 [file pharmaceuticals-13-00270-s001.pdf]

Supplementary Materials to

Structure, antioxidant, and anti-inflammatory activities of the (4*R*)- and (4*S*)-epimers of *S*-carboxymethyl-L-cysteine sulfoxide

James K. Waters, Steven P. Kelley, Valeri V. Mossine, and Thomas P. Mawhinney

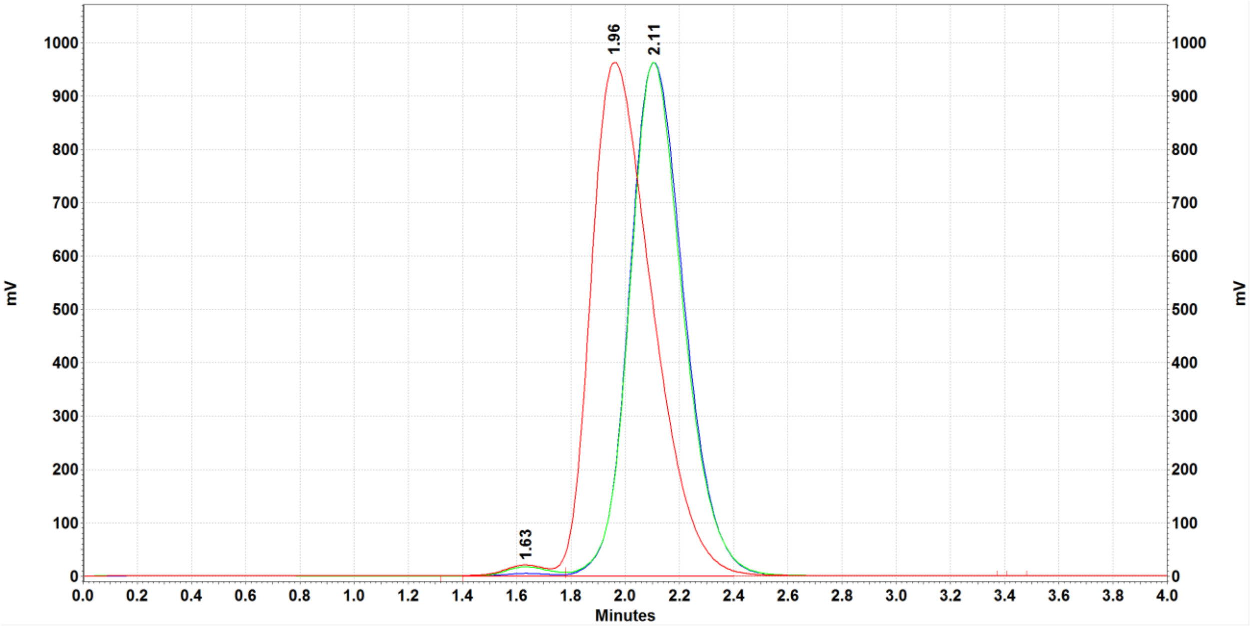

Figure S1. Ion-exchange chromatograms of (2*R*,4*S*)-*S*-carboxymethylcysteine sulfoxide from the monoclinic crystalline material (red), (2*R*,4*R*)-*S*-carboxymethylcysteine sulfoxide from the triclinic crystalline material (green), and the orthorhombic crystalline material (blue, essentially overlaps with green).

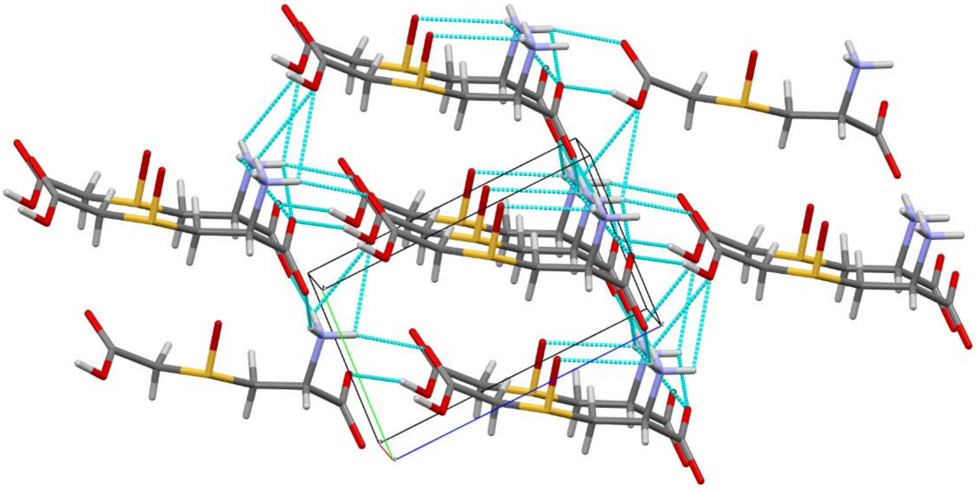

Figure S2. The molecular packing in t-(4*R*)-CMCO. A view of the unit-cell contents shown in projection down the ***a*** axis. Color code for crystallographic axes: red - *a*, green - *b*, blue - *c*. Hydrogen bonds are shown as cyan dotted lines.

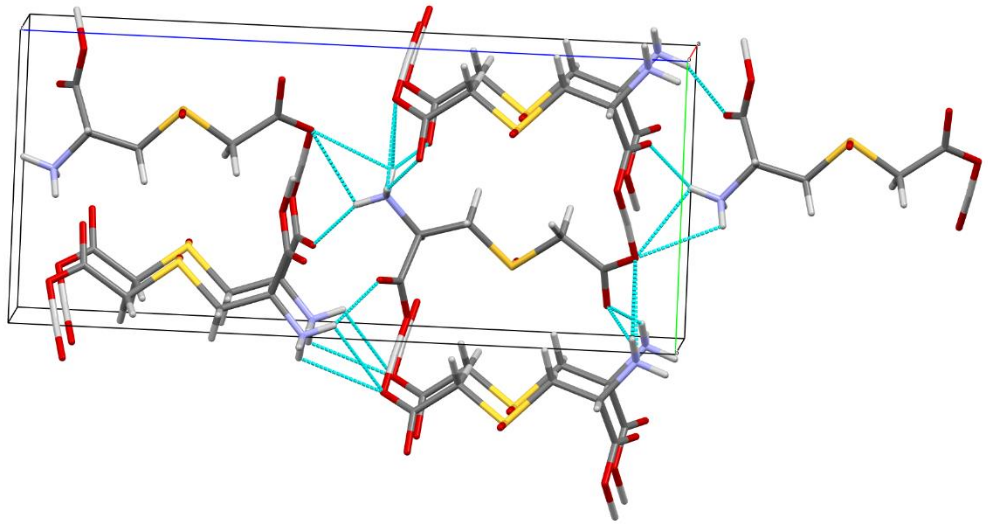

Figure S3. The molecular packing in o-(4*R*)-CMCO. A view of the unit-cell contents shown in projection down the ***a*** axis. Color code for crystallographic axes: red - *a*, green - *b*, blue - *c*. Hydrogen bonds are shown as cyan dotted lines.

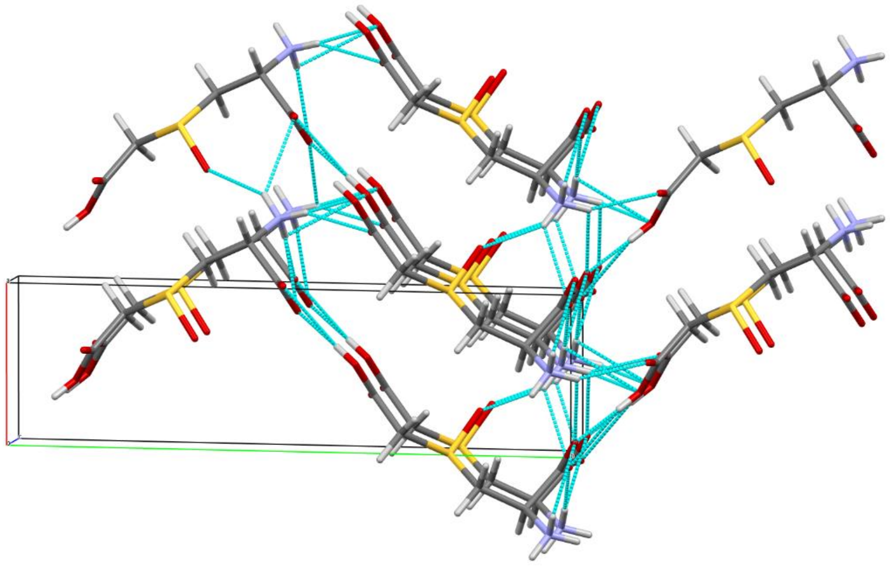

Figure S4. The molecular packing in m-(4*S*)-CMCO. A view of the unit-cell contents shown in projection down the ***c*** axis. Color code for crystallographic axes: red - *a*, green - *b*, blue - *c*. Hydrogen bonds are shown as cyan dotted lines.

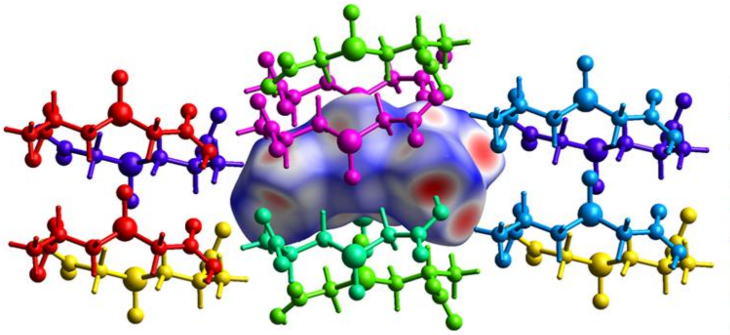

Pairwise interactions energies (kJ/mol)
B3LYP/6-31G(d,p) electron densities energy model

Color code	N	Symm operator	E elstat	E polar	E disp	E repuls	E total
	2	x+1/2,y, -z+1/2	-10.5	-1.1	-2.7	0.5	-13.9
	2	1/2-x,-1/2+y,1/2+z	-4.4	-2	-1.6	0	-7.6
	2	x-1,y,z	42.1	-27.2	-26.1	33.1	22.1
	2	-x,-1/2+y,1/2-z	-159	-51.6	-36	200.5	-113.7
	2	1/2+x,3/2-y,1-z	-144.9	-35.1	-16	54.8	-159.2
	2	1/2-x,1-y,1/2+z	-3.8	-8.9	-8.2	6.1	-13.9
	2	1-x,-1/2+y,1/2-z	-56.3	-22.8	-26.7	55.2	-65.6

Figure S5. Interaction energies in crystal structure of o-(4*R*)-CMCO. Left, a view of interactions between a central (4*R*)-CMCO molecule, shown as its Hirshfeld surface, and 14 molecules that share the interaction surfaces with the central molecule. Right, calculated energies (electrostatic, polarization, dispersion, repulsion, and total) of pairwise interactions in o-(4*R*)-CMCO between the central molecule and those indicated by respective colors.

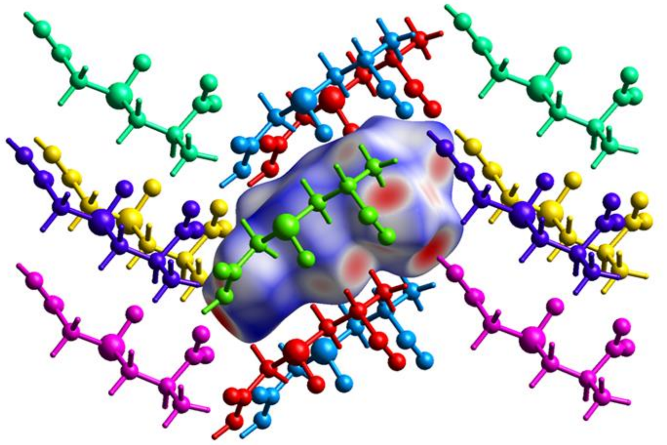

Pairwise interactions energies (kJ/mol)
B3LYP/6-31G(d,p) electron densities energy model

Color code	N	Symm operator	E elstat	E polar	E disp	E repuls	E total
	2	-1+x,y,-1+z	-119.9	-30.1	-21.7	46.7	-139.2
	2	2-x,1/2+y,-z	-10.6	-7.9	-10.3	8.3	-20.9
	2	x,y,-1+z	-29.1	-38.8	-25.7	71.6	-37.7
	2	x-1,y+1/2,-z	-0.8	-2.5	-1.9	0	-4.3
	2	x-1,y,z	7.9	-25.3	-31.4	28.4	-20.2
	2	2-x,1/2+y,1-z	-52.4	-22.1	-19	32.8	-68.1
	2	3-x,-1/2+y,1-z	-118.5	-31	-8.7	130.6	-75.2

Figure S6. Interaction energies in crystal structure of m-(4*S*)-CMCO. Left, a view of interactions between a central (4*S*)-CMCO molecule, shown as its Hirshfeld surface, and 14 molecules that share the interaction surfaces with the central molecule. Right, calculated energies (electrostatic, polarization, dispersion, repulsion, and total) of pairwise interactions in m-(4*S*)-CMCO between the central molecule and those indicated by respective colors.

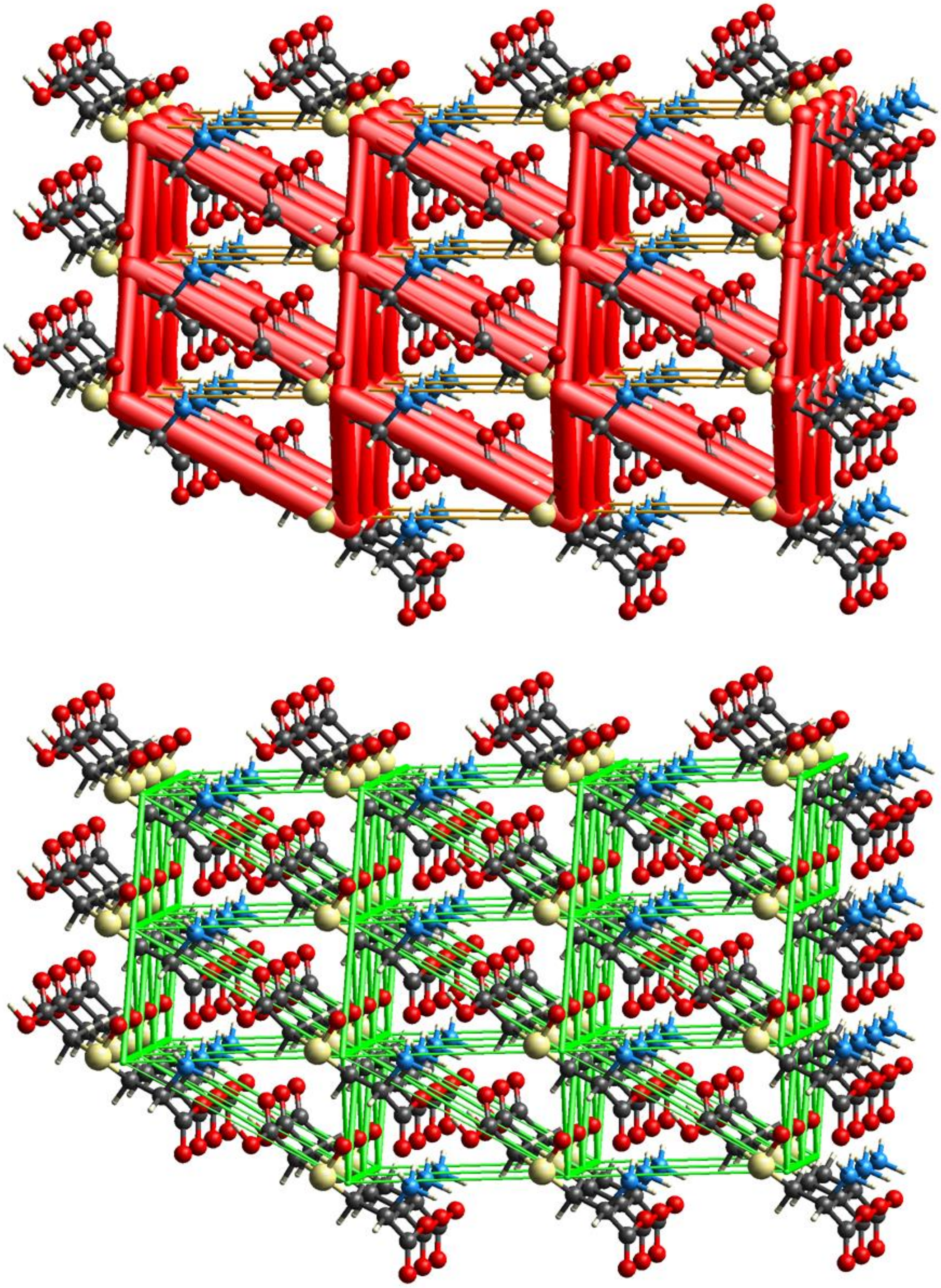

Figure S7. Energy framework for electrostatic (top) and dispersion (bottom) pairwise interaction energies in t-(4*R*)-CMCO. The cylinders link molecular centroids, and the cylinder thickness is proportional to the magnitude of the energies, such as those shown in the Figure 3(b). For clarity, the cylinders corresponding to energies <5 kJ mol-1 are not shown.

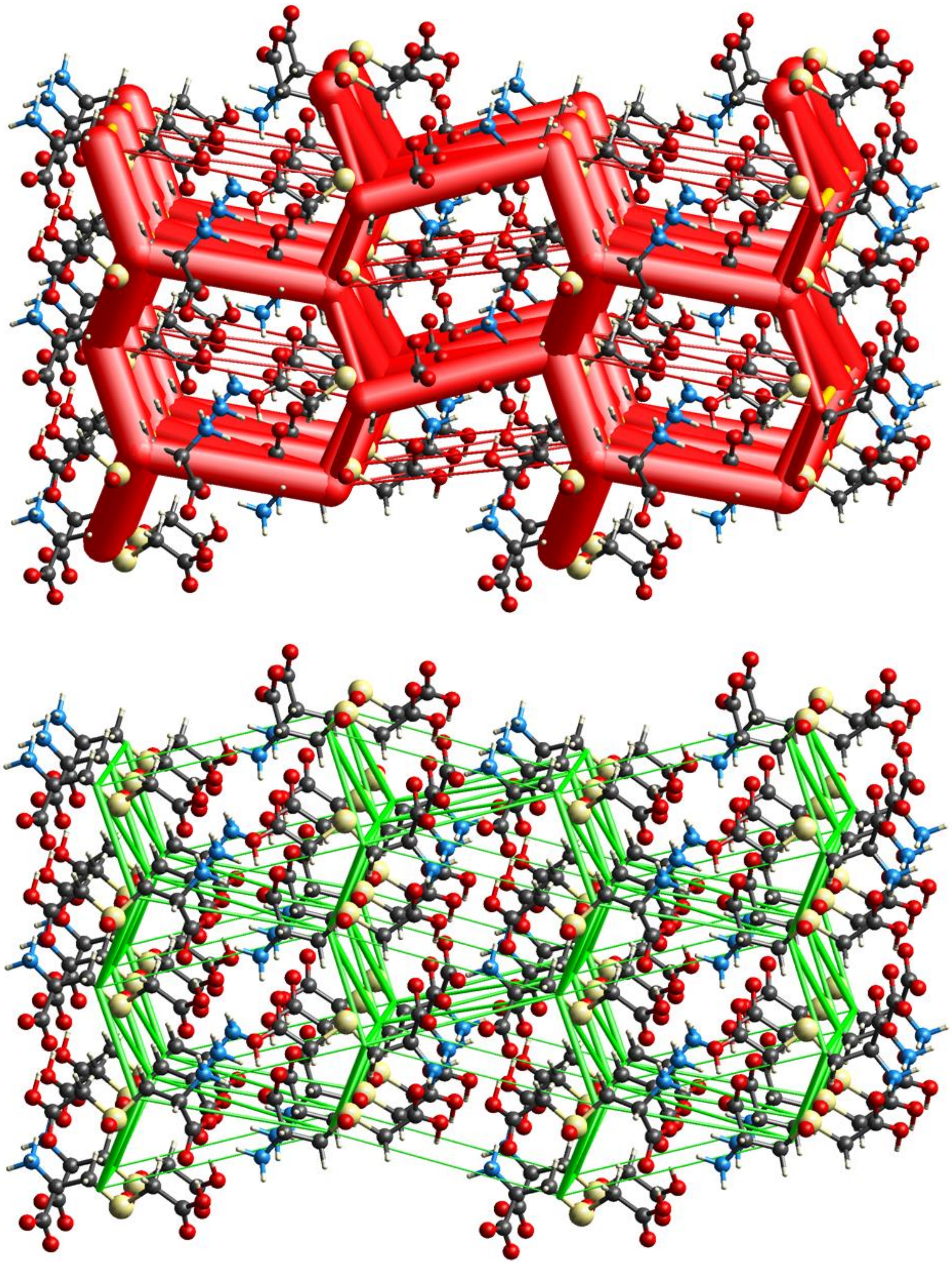

Figure S8. Energy framework for electrostatic (top) and dispersion (bottom) pairwise interaction energies in o-(4*R*)-CMCO. The cylinders link molecular centroids, and the cylinder thickness is proportional to the magnitude of the energies, such as those shown in the Figure S5. For clarity, the cylinders corresponding to energies <5 kJ mol-1 are not shown.

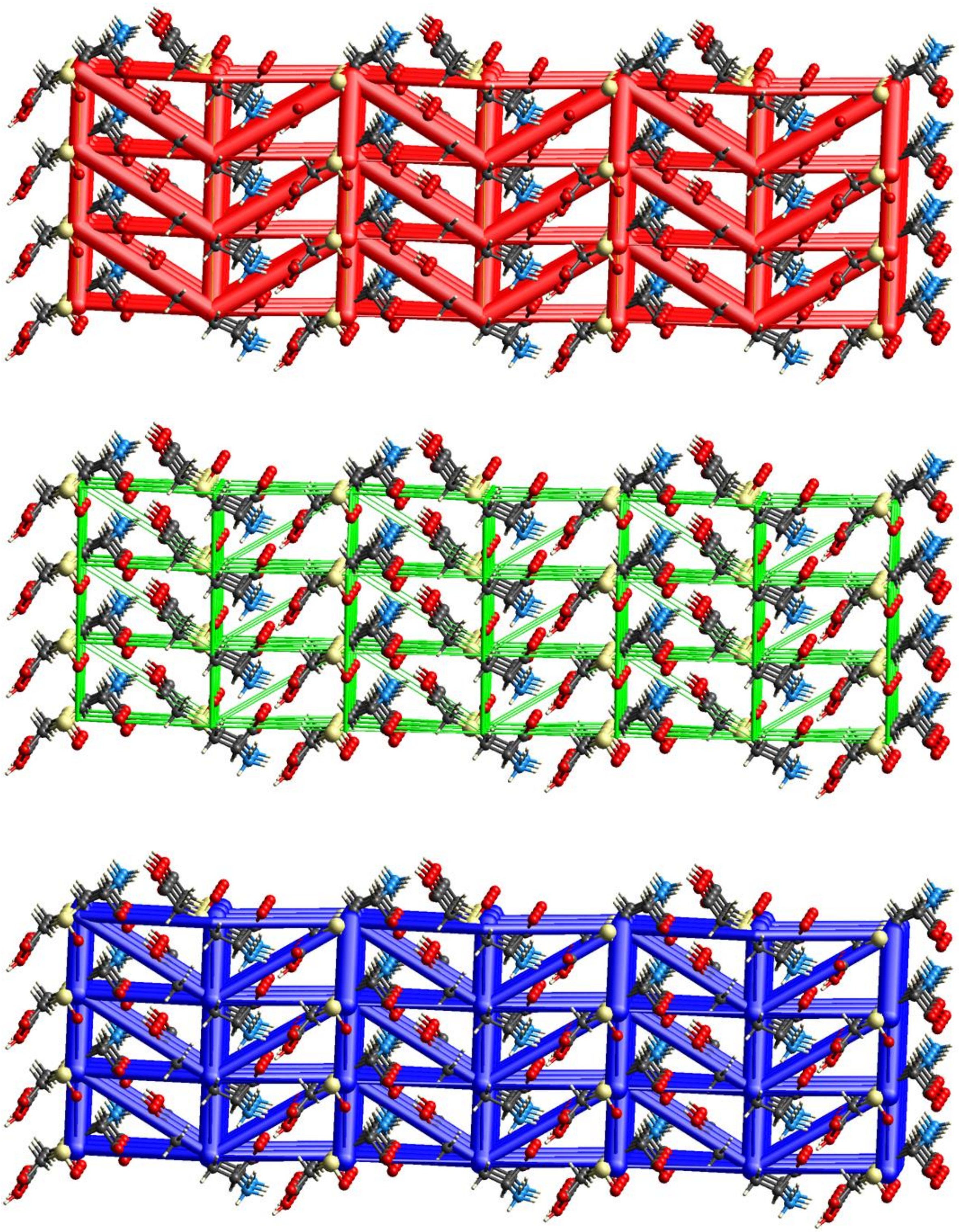

Figure S9. Energy framework for electrostatic (top), dispersion (middle), and total (bottom) pairwise interaction energies in m-(4*S*)-CMCO. The cylinders link molecular centroids, and the cylinder thickness

is proportional to the magnitude of the energies, such as those shown in the Figure S6. For clarity, the cylinders corresponding to energies <5 kJ mol-1 are not shown.

Table S1. Crystallographic parameters of CMCO epimers

Molecule	t-(4*R*)-CMCO	o-(4*R*)-CMCO	m-(4*S*)-CMCO
Crystal data			
Chemical formula	$C_5H_9NO_5S$	$C_5H_9NO_5S$	$C_5H_9NO_5S$
M_r	195.19	195.19	195.19
Crystal system, space group	Triclinic, *P*1	Orthorhombic, $P2_12_12_1$	Monoclinic, $P2_1$
Temperature (K)	100	100	100
a, *b*, *c* (Å)	4.7029 (3), 5.0418 (4), 8.1330 (6)	4.7405 (2), 8.2949 (3), 18.8368 (7)	4.8404 (1), 15.9217 (4), 5.0074 (1)
α, β, γ (°)	85.3347 (18), 87.2699 (17), 74.7011 (18)	90, 90, 90	90, 106.8148 (13), 90
V (Å^3)	185.32 (2)	740.70 (5)	369.41 (1)
Z	1	4	2
Radiation type	Mo *K*α	Cu *K*α	Cu *K*α
μ (mm^{-1})	0.42	3.84	3.85
Crystal size (mm)	0.24 × 0.21 × 0.16	0.7 × 0.05 × 0.05	0.4 × 0.2 × 0.08
Data collection			
Diffractometer	Bruker *APEX* II area detector	Bruker *APEX* II area detector	Bruker *APEX* II area detector
Absorption correction	Multi-scan Bruker AXScale	Multi-scan Bruker AXScale	Multi-scan Bruker AXScale
T_{min}, T_{max}	0.690, 0.747	0.610, 0.754	0.591, 0.754
No. of measured, independent and observed [$I > 2\sigma(I)$] reflections	15283, 3615, 3599	11677, 1463, 1452	4903, 1411, 1410
R_{int}	0.014	0.021	0.022
$(\sin \theta/\lambda)_{max}$ (Å^{-1})	0.839	0.618	0.623
Refinement			
$R[F^2 > 2\sigma(F^2)]$, $wR(F^2)$, S	0.021, 0.058, 1.09	0.022, 0.057, 1.08	0.022, 0.059, 1.07
No. of reflections	3615	1463	1411
No. of parameters	136	121	121

No. of restraints	3	0	4
H-atom treatment	Only H-atom coordinates refined	H atoms treated by a mixture of independent and constrained refinement	H atoms treated by a mixture of independent and constrained refinement
$\Delta\rangle_{max}$, $\Delta\rangle_{min}$ (e Å^{-3})	0.67, -0.18	0.32, -0.28	0.26, -0.17
Absolute structure	Flack x determined using 1781 quotients [(I+)-(I-)]/[(I+)+(I-)] (Parsons, Flack and Wagner, Acta Cryst. B69 (2013) 249-259).	Flack x determined using 568 quotients [(I+)-(I-)]/[(I+)+(I-)]	Flack x determined using 652 quotients [(I+)-(I-)]/[(I+)+(I-)]
Absolute structure parameter	-0.001 (13)	-0.005 (7)	0.007 (9)

Table S2. Fractional atomic coordinates and isotropic or equivalent isotropic displacement parameters (Å^2) for triclinic (2R,4R)-S-carboxymethylcysteine

	x	*y*	*z*	U_{iso}*/U_{eq}
S1	0.66715 (3)	0.79186 (3)	0.55472 (3)	0.00941 (6)
O1	1.25146 (19)	0.37999 (18)	1.00232 (11)	0.01041 (14)
O3	0.4973 (2)	1.0315 (2)	0.64708 (12)	0.01618 (17)
O2	1.08201 (19)	0.02392 (17)	0.94703 (12)	0.01091 (14)
O5	0.5234 (2)	1.05478 (19)	0.22554 (12)	0.01207 (15)
H5	0.415 (6)	1.193 (5)	0.162 (3)	0.018*
O4	0.7702 (2)	1.36728 (19)	0.26535 (12)	0.01313 (15)
N1	0.6845 (2)	0.69944 (19)	0.95603 (12)	0.00895 (15)
H1A	0.670 (6)	0.627 (5)	1.056 (3)	0.013*
H1B	0.782 (5)	0.817 (5)	0.958 (3)	0.013*
H1C	0.514 (6)	0.785 (5)	0.928 (3)	0.013*
C2	0.8233 (2)	0.4776 (2)	0.84389 (14)	0.00783 (16)
H2	0.675 (5)	0.397 (5)	0.817 (3)	0.009*
C1	1.0728 (2)	0.2733 (2)	0.93860 (13)	0.00765 (15)
C3	0.9543 (2)	0.5951 (2)	0.68901 (13)	0.00935 (16)
H3A	1.062 (5)	0.453 (5)	0.628 (3)	0.011*
H3B	1.083 (5)	0.739 (5)	0.725 (3)	0.011*
C5	0.7253 (2)	1.1404 (2)	0.29311 (13)	0.00949 (16)
C4	0.9091 (3)	0.9253 (2)	0.41180 (14)	0.01116 (17)

H4A	1.035 (6)	0.987 (5)	0.478 (3)	0.013*
H4B	1.005 (6)	0.780 (5)	0.361 (3)	0.013*

Table S3. Atomic displacement parameters (Å^2) for triclinic (2R,4R)-S-carboxymethylcysteine

	U^{11}	U^{22}	U^{33}	U^{12}	U^{13}	U^{23}
S1	0.00832 (10)	0.01113 (10)	0.00808 (10)	-0.00188 (7)	-0.00120 (7)	0.00180 (7)
O1	0.0098 (3)	0.0096 (3)	0.0125 (3)	-0.0039 (2)	-0.0036 (3)	0.0015 (2)
O3	0.0147 (4)	0.0161 (4)	0.0126 (4)	0.0043 (3)	0.0016 (3)	0.0002 (3)
O2	0.0105 (3)	0.0065 (3)	0.0156 (4)	-0.0020 (2)	-0.0014 (3)	0.0004 (2)
O5	0.0134 (3)	0.0111 (4)	0.0122 (4)	-0.0042 (3)	-0.0048 (3)	0.0024 (3)
O4	0.0168 (4)	0.0106 (3)	0.0124 (4)	-0.0047 (3)	-0.0017 (3)	0.0015 (3)
N1	0.0084 (3)	0.0084 (3)	0.0094 (4)	-0.0013 (3)	-0.0002 (3)	0.0002 (3)
C2	0.0084 (4)	0.0069 (4)	0.0078 (4)	-0.0015 (3)	-0.0006 (3)	-0.0004 (3)
C1	0.0079 (4)	0.0076 (3)	0.0073 (4)	-0.0022 (3)	-0.0002 (3)	0.0009 (3)
C3	0.0087 (4)	0.0099 (4)	0.0083 (4)	-0.0010 (3)	-0.0003 (3)	0.0014 (3)
C5	0.0100 (4)	0.0099 (4)	0.0078 (4)	-0.0014 (3)	0.0002 (3)	0.0003 (3)
C4	0.0094 (4)	0.0131 (4)	0.0101 (4)	-0.0024 (3)	-0.0011 (3)	0.0031 (3)

Table S4. Geometric parameters (Å, º) for triclinic (2R,4R)-S-carboxymethylcysteine

S1—O3	1.4978 (10)	O4—C5	1.2186 (14)
S1—C3	1.8050 (11)	N1—C2	1.4942 (14)
S1—C4	1.8027 (11)	C2—C1	1.5329 (15)
O1—C1	1.2607 (13)	C2—C3	1.5213 (16)
O2—C1	1.2430 (13)	C5—C4	1.5064 (16)
O5—C5	1.3020 (15)	O5—H5	0.89(2)
N1—H1A	0.87(2)	C2—H2	0.94 (2)
N1—H1B	0.84(2)	C3—H3A	0.93 (2)
N1—H1C	0.84(3)	C3—H3B	1.12 (2)
C4—H4A	0.94 (3)	C4—H4B	0.88 (2)
O3—S1—C3	106.42 (5)	O2—C1—O1	126.09 (10)
O3—S1—C4	105.83 (6)	O2—C1—C2	118.83 (9)
C4—S1—C3	95.65 (5)	C2—C3—S1	110.83 (7)

N1—C2—C1	107.03 (8)	O5—C5—C4	112.86 (10)
N1—C2—C3	110.96 (9)	O4—C5—O5	125.42 (11)
C3—C2—C1	108.68 (9)	O4—C5—C4	121.72 (11)
O1—C1—C2	115.08 (9)	C5—C4—S1	108.92 (8)
C5—O5—H5	108.9 (18)	C2—N1—H1A	109.7 (16)
C2—N1—H1B	112.3(16)	C2—N1—H1C	112.1 (17)
H1A—N1—H1B	109 (2)	H1A—N1—H1C	107 (3)
H1B—N1—H1C	107 (2)	N1—C2—H2	107.0 (15)
C1—C2—H2	112.5 (15)	C3—C2—H2	110.7 (15)
S1—C3—H3A	105.1 (15)	S1—C3—H3B	107.5 (12)
C2—C3—H3A	109.9 (15)	C2—C3—H3B	109.1 (12)
H3A—C3—H3B	114.4 (19)	S1—C4—H4A	105.4 (15)
S1—C4—H4B	103.2 (17)	C5—C4—H4A	115.9 (15)
C5—C4—H4B	111.0 (16)	H4A—C4—H4B	112 (2)
O3—S1—C3—C2	63.94(8)	C4—S1—C3—C2	172.29(7)
O3—S1—C4—C5	-62.50(8)	C3—S1—C4—C5	-171.35(7)
O1—C1—C2—N1	-51.18(12)	O1—C1—C2—C3	68.72(12)
O2—C1—C2—N1	128.46(10)	O2—C1—C2—C3	-111.64(11)
N1—C2—C3—S1	-72.24(9)	C1—C2—C3—S1	170.34(7)
S1—C4—C5—O4	129.92(10)	S1—C4—C5—O5	-50.83(11)
O3—S1—C3—H3A	-177.4(16)	O3—S1—C3—H3B	-55.2(13)
C4—S1—C3—H3A	-69.0(16)	C4—S1—C3—H3B	53.2(13)
O3—S1—C4—H4A	62.5(16)	O3—S1—C4—H4B	179.6(18)
C3—S1—C4—H4A	-46.3(16)	C3—S1—C4—H4B	70.7(18)
H5—O5—C5—O4	-4.4(18)	H5—O5—C5—C4	176.4(18)
O1—C1—C2—H2	-168.4(16)	O2—C1—C2—H2	11.3(16)
C1—C2—N1—H1A	-41(2)	C1—C2—N1—H1B	80.1(18)
C1—C2—N1—H1C	-159.5(19)	C3—C2—N1—H1A	-159(2)
C3—C2—N1—H1B	-38.3(18)	C3—C2—N1—H1C	82.1(19)
H2—C2—N1—H1A	80(3)	H2—C2—N1—H1B	-159(2)
H2—C2—N1—H1C	-39(2)	N1—C2—C3—H3A	172.0(16)
N1—C2—C3—H3B	45.9(13)	C1—C2—C3—H3A	54.6(16)
C1—C2—C3—H3B	-71.5(13)	H2—C2—C3—S1	46.3(16)
H2—C2—C3—H3A	-69(2)	H2—C2—C3—H3B	165(2)
H4A—C4—C5—O4	11.3(18)	H4A—C4—C5—O5	-169.4(18)
H4B—C4—C5—O4	-117(2)	H4B—C4—C5—O5	62(2)

Table S5. Hydrogen-bond geometry (Å, º) for triclinic (2R,4R)-S-carboxymethylcysteine

D*-H ··· *A	***D*-H**	**H ··· *A***	***D* ··· *A***	***D*-H ··· *A***	**Symmetry**
N1—H1A ··· O4	0.87 (2)	2.06 (2)	2.8898 (14)	160 (2)	x, -1+y, 1+z
N1—H1A ··· O5	0.87 (2)	2.58 (2)	2.8901 (14)	102.3 (18)	x, y, 1+z
N1—H1B ··· O2	0.84 (2)	1.96 (2)	2.7853 (13)	167 (2)	x, 1+y, z
N1—H1C ··· O2	0.84 (3)	2.08 (3)	2.8748 (13)	159 (2)	-1+x, 1+y, z
N1—H1C ··· O3	0.84 (3)	2.51 (2)	2.9394 (14)	113 (2)	
O5—H5 ··· O1	0.89 (2)	1.64 (3)	2.5001 (13)	163 (2)	-1+x,1+y,-1+z

Table S6. Fractional atomic coordinates and isotropic or equivalent isotropic displacement parameters (Å^2) for orthorhombic (2R,4R)-S-carboxymethylcysteine

	x	*y*	*z*	U_{iso}*/U_{eq}
S1	0.31093 (10)	0.74495 (5)	0.25360 (2)	0.00996 (14)
O1	-0.0768 (3)	0.79019 (16)	0.44544 (7)	0.0111 (3)
O2	0.2922 (3)	0.93446 (16)	0.40635 (7)	0.0126 (3)
O5	-0.0191 (3)	0.67693 (18)	0.06561 (8)	0.0127 (3)
O4	0.2728 (3)	0.85987 (17)	0.10865 (7)	0.0123 (3)
O3	0.6267 (3)	0.74233 (18)	0.25103 (7)	0.0167 (3)
N1	0.2422 (4)	0.5189 (2)	0.44529 (9)	0.0094 (3)
C1	0.1601 (4)	0.8031 (2)	0.41839 (10)	0.0087 (4)
C5	0.1495 (4)	0.7292 (2)	0.11427 (9)	0.0090 (4)
C3	0.2087 (5)	0.5987 (2)	0.32013 (9)	0.0097 (4)
H3A	0.000813	0.587531	0.320475	0.012*
H3B	0.290859	0.492366	0.308113	0.012*
C4	0.1813 (5)	0.6258 (2)	0.17996 (10)	0.0105 (4)
H4A	0.314524	0.536650	0.170153	0.013*
H4B	-0.003443	0.578159	0.192574	0.013*
C2	0.3104 (5)	0.6505 (2)	0.39383 (10)	0.0090 (4)
H2	0.518739	0.669126	0.392887	0.011*
H1A	0.064 (7)	0.488 (3)	0.4423 (14)	0.013*
H1B	0.345 (6)	0.437 (3)	0.4392 (13)	0.013*
H1C	0.272 (6)	0.556 (3)	0.4899 (14)	0.013*

H5	-0.124 (6)	0.556 (3)	0.0783 (13)	0.013*

Table S7. Atomic displacement parameters (Å^2) for orthorhombic (2R,4R)-S-carboxymethylcysteine

	U^{11}	U^{22}	U^{33}	U^{12}	U^{13}	U^{23}
S1	0.0124 (3)	0.0094 (2)	0.0080 (2)	-0.00188 (18)	-0.00014 (16)	0.00023 (19)
O1	0.0092 (7)	0.0122 (7)	0.0120 (6)	0.0006 (5)	0.0009 (5)	-0.0021 (5)
O2	0.0117 (7)	0.0083 (6)	0.0177 (7)	0.0011 (6)	0.0017 (6)	0.0016 (5)
O5	0.0173 (7)	0.0096 (6)	0.0111 (7)	-0.0031 (6)	-0.0035 (6)	0.0010 (5)
O4	0.0138 (7)	0.0108 (6)	0.0124 (6)	-0.0028 (6)	0.0007 (6)	0.0004 (5)
O3	0.0118 (7)	0.0213 (7)	0.0170 (7)	-0.0031 (6)	-0.0002 (5)	0.0027 (8)
N1	0.0108 (8)	0.0077 (7)	0.0096 (8)	0.0011 (7)	-0.0018 (7)	-0.0004 (6)
C1	0.0107 (9)	0.0103 (9)	0.0051 (8)	0.0015 (8)	-0.0045 (7)	0.0000 (6)
C5	0.0089 (8)	0.0090 (9)	0.0090 (8)	0.0011 (8)	0.0027 (7)	-0.0019 (7)
C3	0.0121 (10)	0.0085 (8)	0.0086 (8)	-0.0003 (8)	0.0003 (8)	0.0000 (6)
C4	0.0132 (10)	0.0096 (8)	0.0087 (9)	-0.0009 (8)	-0.0002 (8)	-0.0004 (7)
C2	0.0090 (9)	0.0086 (8)	0.0093 (9)	0.0003 (8)	0.0007 (8)	0.0006 (7)

Table S8. Geometric parameters (Å, º) for orthorhombic (2R,4R)-S-carboxymethylcysteine

S1—O3	1.4978 (15)	N1—H1B	0.84 (3)
S1—C3	1.8104 (19)	N1—H1C	0.91 (3)
S1—C4	1.8105 (19)	C1—C2	1.525 (3)
O1—C1	1.238 (3)	C5—C4	1.513 (3)
O2—C1	1.277 (2)	C3—H3A	0.99
O5—C5	1.291 (2)	C3—H3B	0.99
O5—H5	1.14 (3)	C3—C2	1.531 (2)
O4—C5	1.236 (2)	C4—H4A	0.99
N1—C2	1.495 (2)	C4—H4B	0.99
N1—H1A	0.88 (3)	C2—H2	1.00
O3—S1—C3	106.27 (9)	S1—C3—H3B	109.5
O3—S1—C4	107.84 (9)	H3A—C3—H3B	108.1
C3—S1—C4	94.23 (8)	C2—C3—S1	110.81 (13)

C5—O5—H5	114.5 (13)	C2—C3—H3A	109.5
C2—N1—H1A	112.0 (17)	C2—C3—H3B	109.5
C2—N1—H1B	111.9 (18)	S1—C4—H4A	109.5
C2—N1—H1C	108.6 (16)	S1—C4—H4B	109.5
H1A—N1—H1B	108 (2)	C5—C4—S1	110.56 (13)
H1A—N1—H1C	108 (2)	C5—C4—H4A	109.5
H1B—N1—H1C	108 (2)	C5—C4—H4B	109.5
O1—C1—O2	126.31 (18)	H4A—C4—H4B	108.1
O1—C1—C2	118.47 (17)	N1—C2—C1	107.96 (16)
O2—C1—C2	115.18 (17)	N1—C2—C3	108.37 (15)
O5—C5—C4	116.88 (17)	N1—C2—H2	109.8
O4—C5—O5	121.76 (17)	C1—C2—C3	111.17 (16)
O4—C5—C4	121.33 (17)	C1—C2—H2	109.8
S1—C3—H3A	109.5	C3—C2—H2	109.8
O3—S1—C3—C2	64.88(16)	C4—S1—C3—C2	174.87(16)
O3—S1—C4—C5	-88.32(16)	C3—S1—C4—C5	163.08(16)
O1—C1—C2—N1	-36.4(2)	O1—C1—C2—C3	82.4(2)
O2—C1—C2—N1	145.90(17)	O2—C1—C2—C3	-95.4(2)
N1—C2—C3—S1	-175.16(14)	C1—C2—C3—S1	66.4(2)
S1—C4—C5—O4	19.1(2)	S1—C4—C5—O5	-158.78(14)
O3—S1—C3—H3A	-174	O3—S1—C3—H3B	-56
C4—S1—C3—H3A	-64	C4—S1—C3—H3B	54
O3—S1—C4—H4A	32	O3—S1—C4—H4B	151
C3—S1—C4—H4A	-76	C3—S1—C4—H4B	42
H5—O5—C5—O4	-178.3(15)	H5—O5—C5—C4	-0.5(15)
O1—C1—C2—H2	-156	O2—C1—C2—H2	26
C1—C2—N1—H1A	69.9(19)	C1—C2—N1—H1B	-168.4(19)
C1—C2—N1—H1C	-49.2(19)	C3—C2—N1—H1A	-50.6(19)
C3—C2—N1—H1B	71.1(19)	C3—C2—N1—H1C	-169.7(19)
H2—C2—N1—H1A	170	H2—C2—N1—H1B	-49
H2—C2—N1—H1C	70	N1—C2—C3—H3A	64
N1—C2—C3—H3B	-54	C1—C2—C3—H3A	-55
C1—C2—C3—H3B	-173	H2—C2—C3—S1	-55
H2—C2—C3—H3A	-176	H2—C2—C3—H3B	66
H4A—C4—C5—O4	-102	H4A—C4—C5—O5	80
H4B—C4—C5—O4	140	H4B—C4—C5—O5	-38

Table S9. Hydrogen-bond geometry (Å, º) for orthorhombic (2R,4R)-S-carboxymethylcysteine

D*-H ··· *A	***D*-H**	**H ··· *A***	***D* ··· *A***	***D*-H ··· *A***	**Symmetry**
N1—H1A ··· O4	0.89 (3)	2.15 (3)	2.955 (2)	152 (2)	-x, -1/2+y, 1/2-z
N1—H1A ··· O5	0.89 (3)	2.59 (3)	3.034 (2)	112 (2)	-x, -1/2+y, 1/2-z
N1—H1B ··· O4	0.84 (3)	2.12 (3)	2.839 (2)	143 (2)	1-x, -1/2+y, 1/2-z
N1—H1C ··· O1	0.91 (3)	1.90 (3)	2.735 (2)	152 (2)	1/2+x, 3/2-y, 1-z
O5—H5 ··· O1	1.15 (3)	2.44 (3)	3.247 (2)	125.7 (19)	-x, -1/2+y, 1/2-z
O5—H5 ··· O2	1.15 (3)	1.32 (3)	2.450 (2)	168 (3)	-x, -1/2+y, 1/2-z

Table S10. Fractional atomic coordinates and isotropic or equivalent isotropic displacement parameters (Å^2) for monoclinic (2R,4S)-S-carboxymethylcysteine

	x	*y*	*z*	U_{iso}*/U_{eq}
S1	1.03017 (12)	0.79592 (4)	0.47269 (11)	0.01146 (17)
O1	0.9543 (4)	1.00388 (11)	0.6725 (3)	0.0129 (4)
O4	1.3720 (4)	0.65219 (11)	0.7361 (4)	0.0157 (4)
O2	1.1217 (4)	1.02197 (11)	0.3051 (4)	0.0131 (4)
C1	0.9482 (5)	0.99206 (14)	0.4247 (5)	0.0103 (5)
N1	0.5682 (5)	0.98290 (15)	-0.0173 (4)	0.0123 (4)
O3	1.2910 (4)	0.84677 (11)	0.6187 (4)	0.0176 (4)
O5	1.5730 (4)	0.63054 (11)	0.3903 (4)	0.0137 (4)
C2	0.7112 (5)	0.93523 (15)	0.2423 (5)	0.0101 (5)
H2	0.567192	0.920922	0.343697	0.012*
C5	1.3819 (6)	0.66260 (15)	0.4978 (5)	0.0119 (5)
C4	1.1600 (5)	0.71471 (15)	0.2877 (5)	0.0132 (5)
H4A	0.997529	0.678654	0.184364	0.016*
H4B	1.248351	0.740277	0.151824	0.016*
C3	0.8412 (5)	0.85451 (15)	0.1612 (5)	0.0117 (5)
H3B	0.977270	0.869022	0.053954	0.014*
H3A	0.685578	0.819344	0.041106	0.014*
H1A	0.527 (7)	1.0306 (18)	0.041 (6)	0.018*
H1B	0.432 (6)	0.955 (2)	-0.124 (7)	0.018*
H1C	0.681 (7)	0.991 (2)	-0.113 (7)	0.018*

H5	1.672 (8)	0.596 (2)	0.496 (8)	0.018*

Table S11. Atomic displacement parameters (Å^2) for monoclinic (2R,4S)-S-carboxymethylcysteine

	U^{11}	U^{22}	U^{33}	U^{12}	U^{13}	U^{23}
S1	0.0135 (3)	0.0109 (3)	0.0092 (3)	0.0013 (2)	0.0021 (2)	-0.0003 (2)
O1	0.0139 (9)	0.0168 (9)	0.0078 (8)	-0.0003 (7)	0.0026 (6)	-0.0010 (7)
O4	0.0220 (9)	0.0153 (8)	0.0103 (9)	0.0032 (7)	0.0056 (7)	0.0017 (7)
O2	0.0138 (9)	0.0161 (9)	0.0101 (8)	-0.0033 (7)	0.0046 (7)	-0.0019 (7)
C1	0.0108 (11)	0.0095 (10)	0.0092 (10)	0.0036 (9)	0.0007 (9)	0.0008 (9)
N1	0.0120 (10)	0.0157 (11)	0.0082 (10)	0.0008 (8)	0.0013 (8)	0.0005 (8)
O3	0.0177 (9)	0.0128 (8)	0.0168 (9)	0.0015 (7)	-0.0038 (7)	-0.0007 (7)
O5	0.0144 (9)	0.0156 (9)	0.0115 (9)	0.0044 (7)	0.0043 (7)	0.0012 (7)
C2	0.0101 (11)	0.0113 (11)	0.0077 (11)	0.0011 (9)	0.0008 (9)	0.0010 (9)
C5	0.0143 (12)	0.0092 (11)	0.0118 (12)	-0.0020 (9)	0.0032 (9)	-0.0007 (9)
C4	0.0144 (11)	0.0139 (10)	0.0111 (11)	0.0031 (9)	0.0032 (9)	-0.0005 (9)
C3	0.0115 (11)	0.0128 (11)	0.0093 (11)	0.0006 (9)	0.0006 (9)	-0.0002 (9)

Table S12. Geometric parameters (Å, º) for monoclinic (2R,4S)-S-carboxymethylcysteine

S1—O3	1.500 (2)	C1—C2	1.537 (3)
S1—C4	1.806 (3)	N1—C2	1.492 (3)
S1—C3	1.821 (2)	O5—C5	1.302 (3)
O1—C1	1.247 (3)	C2—C3	1.536 (3)
O4—C5	1.219 (3)	C5—C4	1.515 (3)
O2—C1	1.257 (3)	O5—H5	0.82(4)
N1—H1A	0.86(3)	C2—H2	1.00
N1—H1B	0.85(3)	C3—H3A	0.99
N1—H1C	0.83(3)	C3—H3B	0.99
C4—H4A	0.99	C4—H4B	0.99
O3—S1—C4	105.54 (12)	N1—C2—C3	108.50 (18)
O3—S1—C3	106.54 (11)	C3—C2—C1	110.9 (2)
C4—S1—C3	95.19 (11)	O4—C5—O5	125.8 (2)

O1—C1—O2	126.2 (2)	O4—C5—C4	122.2 (2)
O1—C1—C2	118.6 (2)	O5—C5—C4	111.9 (2)
O2—C1—C2	115.2 (2)	C5—C4—S1	108.53 (17)
N1—C2—C1	107.26 (19)	C2—C3—S1	110.16 (15)
C5—O5—H5	111 (3)	C2—N1—H1A	104.4 (19)
C2—N1—H1B	111 (2)	C2—N1—H1C	111 (2)
H1A—N1—H1B	118 (3)	H1A—N1—H1C	108 (3)
H1B—N1—H1C	104 (3)	N1—C2—H2	110
C1—C2—H2	110	C3—C2—H2	110
S1—C3—H3A	110	S1—C3—H3B	110
C2—C3—H3A	110	C2—C3—H3B	110
H3A—C3—H3B	108	S1—C4—H4A	110
S1—C4—H4B	110	C5—C4—H4A	110
C5—C4—H4B	110	H4A—C4—H4B	108
O3—S1—C3—C2	-68.68 (19)	C4—S1—C3—C2	-176.59 (18)
O3—S1—C4—C5	60.08 (19)	C3—S1—C4—C5	168.85 (18)
O1—C1—C2—N1	128.3 (2)	O1—C1—C2—C3	-113.3 (2)
O2—C1—C2—N1	-52.3 (3)	O2—C1—C2—C3	66.1 (3)
N1—C2—C3—S1	177.90(17)	C1—C2—C3—S1	60.3 (2)
S1—C4—C5—O4	30.3 (3)	S1—C4—C5—O5	-150.91(18)
O3—S1—C3—H3A	171	O3—S1—C3—H3B	52
C4—S1—C3—H3A	63	C4—S1—C3—H3B	-56
O3—S1—C4—H4A	-180	O3—S1—C4—H4B	-60
C3—S1—C4—H4A	-71	C3—S1—C4—H4B	48
H5—O5—C5—O4	9 (3)	H5—O5—C5—C4	-170 (3)
O1—C1—C2—H2	9	O2—C1—C2—H2	-172
C1—C2—N1—H1A	-50 (2)	C1—C2—N1—H1B	-178 (2)
C1—C2—N1—H1C	66 (2)	C3—C2—N1—H1A	-170(2)
C3—C2—N1—H1B	62(2)	C3—C2—N1—H1C	-54(2)
H2—C2—N1—H1A	69	H2—C2—N1—H1B	-59
H2—C2—N1—H1C	-174	N1—C2—C3—H3A	-61
N1—C2—C3—H3B	57	C1—C2—C3—H3A	-179
C1—C2—C3—H3B	-60	H2—C2—C3—S1	-62
H2—C2—C3—H3A	59	H2—C2—C3—H3B	178
H4A—C4—C5—O4	-90	H4A—C4—C5—O5	89
H4B—C4—C5—O4	151	H4B—C4—C5—O5	-31

Table S13. Hydrogen-bond geometry (Å, º) for monoclinic (2R,4S)-S-carboxymethylcysteine

D*-H ··· *A	***D*-H**	**H ··· *A***	***D* ··· *A***	***D*-H ··· *A***	**Symmetry**
N1—H1A ··· O4	0.86 (3)	2.22 (3)	3.016 (3)	155 (3)	2-x, 1/2+y, 1-z
N1—H1B ··· O1	0.85(3)	2.37 (3)	2.947 (3)	126 (3)	-1+x, y, -1+z
N1—H1B ··· O3	0.85(3)	2.14 (3)	2.899 (3)	149 (3)	-1+x, y, -1+z
N1—H1C ··· O1	0.83(3)	1.94 (3)	2.774 (3)	177 (3)	x, y, -1+z
O5—H5 ··· O2	0.82(4)	1.67 (4)	2.489 (3)	176 (4)	3-x, -1/2+y, 1-z
